# Inulin-g-poly-D,L-lactide, a sustainable amphiphilic copolymer for nano-therapeutics

**DOI:** 10.1007/s13346-022-01135-4

**Published:** 2022-02-22

**Authors:** Carla Sardo, Teresa Mencherini, Carmela Tommasino, Tiziana Esposito, Paola Russo, Pasquale Del Gaudio, Rita Patrizia Aquino

**Affiliations:** Department of Pharmacy, University of Salerno, via Giovanni Paolo II, 132, 84084 Fisciano, SA Italy

**Keywords:** Inulin, PLA, PEG alternative, Nanoparticles, Doxorubicin

## Abstract

Cancer therapies started to take a big advantage from new nanomedicines on the market. Since then, research tried to better understand how to maximize efficacy while maintaining a high safety profile. Polyethylene glycol (PEG), the gold standard for nanomedicines coating design, is a winning choice to ensure a long circulation and colloidal stability, while in some cases, patients could develop PEG-directed immunoglobulins after the first administration. This lead to a phenomenon called accelerated blood clearance (ABC effect), and it is correlated with clinical failure because of the premature removal of the nanosystem from the circulation by immune mechanism. Therefore, alternatives to PEG need to be found. Here, looking at the backbone structural analogy, the hydrophilicity, flexibility, and its GRAS status, the natural polysaccharide inulin (INU) was investigated as PEG alternative. In particular, the first family of Inulin-g-poly-D,L-lactide amphiphilic copolymers (INU-PLAs) was synthesized. The new materials were fully characterized from the physicochemical point of view (solubility, 1D and 2D NMR, FT-IR, UV–Vis, GPC, DSC) and showed interesting hybrid properties compared to precursors. Moreover, their ability in forming stable colloids and to serve as a carrier for doxorubicin were investigated and compared with the already well-known and well-characterized PEGylated counterpart, polyethylene glycol-b-poly-D,L-lactide (PEG-PLA). This preliminary investigation showed INU-PLA to be able to assemble in nanostructures less than 200 nm in size and capable of loading doxorubicin with an encapsulation efficiency in the same order of magnitude of PEG-PLA analogues.

**Graphical abstract:**

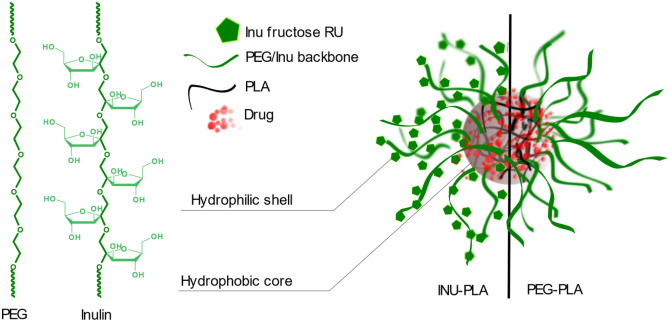

**Supplementary information:**

The online version contains supplementary material available at 10.1007/s13346-022-01135-4.

## Introduction

Most promising cancer nanomedicines are structures where a hydrophobic core serves as a matrix for the homogeneous distribution of lipophilic active substances, while a hydrophilic shell serves as a stabilizing interface between the core and the external aqueous environment [1]. In those structures, referred to as core–shell nanosystems, one of the components is mostly polyethylene glycol (PEG). PEG entered the practice of all labs of nanomaterials for biomedical applications and even the market, as the gold standard among hydrophilic synthetic blocks in copolymers design as the corona-forming block. The biocompatibility, high flexibility, large exclusion volume in water, and easiness of synthesis and supply are the reasons for the majority of literature conferring to PEG the leading role in the design of drug delivery systems. PEG is considered particularly useful as colloidal stabilizer and to prevent the adsorption of proteins, adhesion of cells, and the consequent premature blood clearance [2–5]. Nevertheless, there are some disadvantages one should consider. First, PEG is not a naturally occurring polymer, and it is synthesized via exothermic reaction on ethylene oxide. Second, the existence of antibody-mediated anti-PEG immunity [6], which can lead to the “accelerated blood clearance” (ABC effect) of PEGylated therapeutics, has been reported [7, 8]. Although anti-PEG immunity is still being studied in humans and to date the influence of the system and subject variability is not very well defined, the reduced efficacy of PEGylated therapeutics in clinical trials has been related to the ABC effect [9, 10].

In contrast to PEG, inulin (INU) is a natural biocompatible polysaccharide, easily extracted from different vegetable sources. In recent years, INU is growingly used as substrate to obtain advanced functional materials for drug delivery, thanks to the number of hydroxyls of the dangling fructose units which allow its functionalization. For example, INU has already been modified with α-tocopherol, ceramide, squalene, folic acid, and biotin [11–13] to obtain drug delivery vehicles with specific features. Polycationic inulin derivatives have also been obtained and employed as nucleic acid delivery systems [14, 15]. Mandracchia and colleagues, for example, obtained inulin-based nanomicelles circulating up to 48 h after intravenous administration in balb c/mice [16, 17]. In that case, one can imagine that the fast filtration by the kidney typical of INU in its “natural” unmodified soluble conformation could be impaired by the higher hydrodynamic size of nanosystems. A maintained excretion in some extent could provide a way to address organ toxicity associated with repeated dosing of unclearable nanoparticles [18].

As well as PEG, INU is classified under the Generally Recognized As Safe (GRAS status by the United States Food and Drug Administration) since 2002. One of the most peculiar characteristics of INU, compared with other polysaccharides, is that its sugar rings are not incorporated in the backbone, which adds to the hydrophilicity, the good solubility in water and organic solvents, and the uncharged character, a high chain flexibility. Looking closer to the backbone, it is possible to notice that the above-mentioned analogies in the physicochemical characteristics of PEG and INU are also reflected in their structural similarity, since they share the same backbone.

Thus, the question is, can INU be considered a naturally occurring, functionalizable alternative to PEG? To verify this, we synthesized the first Inulin-g-poly-D,L-lactide amphiphilic copolymer (INU-PLA). Its water self-assembling and the ability to serve as a drug carrier (doxorubicin here used as model drug) were investigated and compared with the already well-known and well-characterized PEGylated counterpart, Polyethylene glycol-g-poly-D,L-lactide (PEG-PLA).

The positive answer to that question would break a wall in the biomaterials field, opening to opportunities never considered before, thanks to the alternative it would represent in that cases where a PEG immunity is an obstacle for a successful therapeutic outcome [19, 20], as well as for the structural variety of the derivatives that could stem from considering INU a valid not immunogenic [21, 22] alternative to PEG.

## Results and discussion

### Synthesis of INU-PLA copolymers

**Fig. 1:**
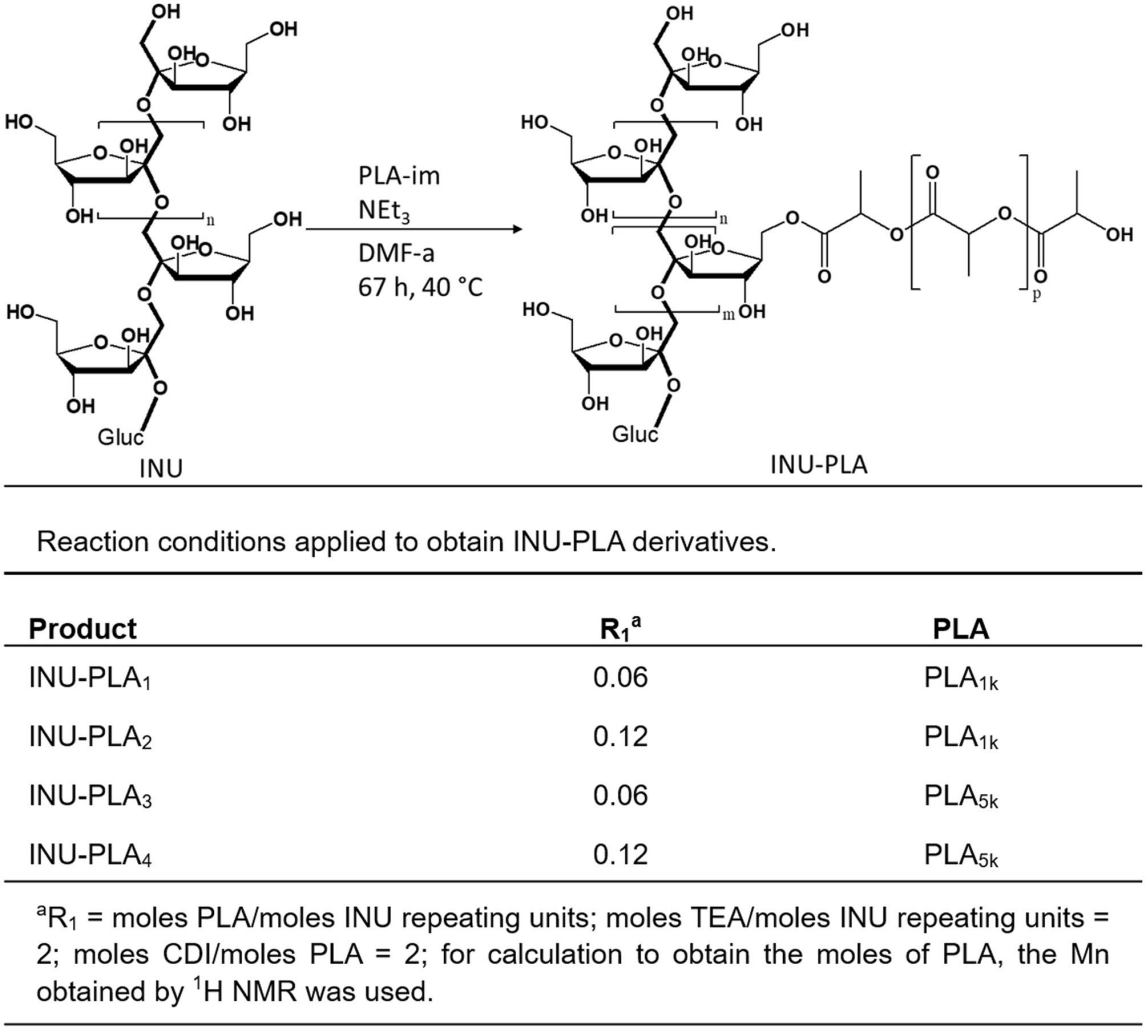
Scheme of the synthesis and reaction conditions to obtain INU-PLA 1–4

The synthesis of INU-PLA copolymers was carried out in very mild conditions (Fig. 1). By using CDI as coupling agent, the imidazolide derivative of PLA (PLA-im) can be obtained [23] and used in one pot alcoholysis by adding INU, previously treated with a base. CDI has been preferred over other activating agents, such as DCC, because pure water hydrolyzes it at room temperature in short time, with evolution of CO_2_, and the reaction gives imidazole as the sole side product. The water solubility and non-toxic nature of such compound represent a good opportunity for a green option, coherently with the choice of INU as a renewable substrate. The purification from unreacted PLA was carried out by means of precipitation and washings in diethyl ether (EtOEt). Since PLA_5k_ has a lower solubility in EtOEt compared to PLA_1k_, 15% of acetone was added to completely eliminate any unreacted PLA. Considering earlier literature [24] and predictable reactivity, it is likely that functionalization occurs at the primary hydroxyls of inulin with the activated end-carboxylic acid group of PLA.

Four derivatives, named INU-PLA_1-4_, were synthesized by using two different PLA with Mn of 1041 Da and 5017 Da, here named PLA_1k_ and PLA_5k_, respectively. In turn, those two polyesters were grafted onto INU by applying two different molar ratios between PLA and INU repeating units (R_1_), i.e., 0.06 and 0.12. The reaction conditions and the names attributed to the four obtained derivatives are summarized in Fig. 1.

### Characterization of INU-PLA copolymers

**Fig. 2:**
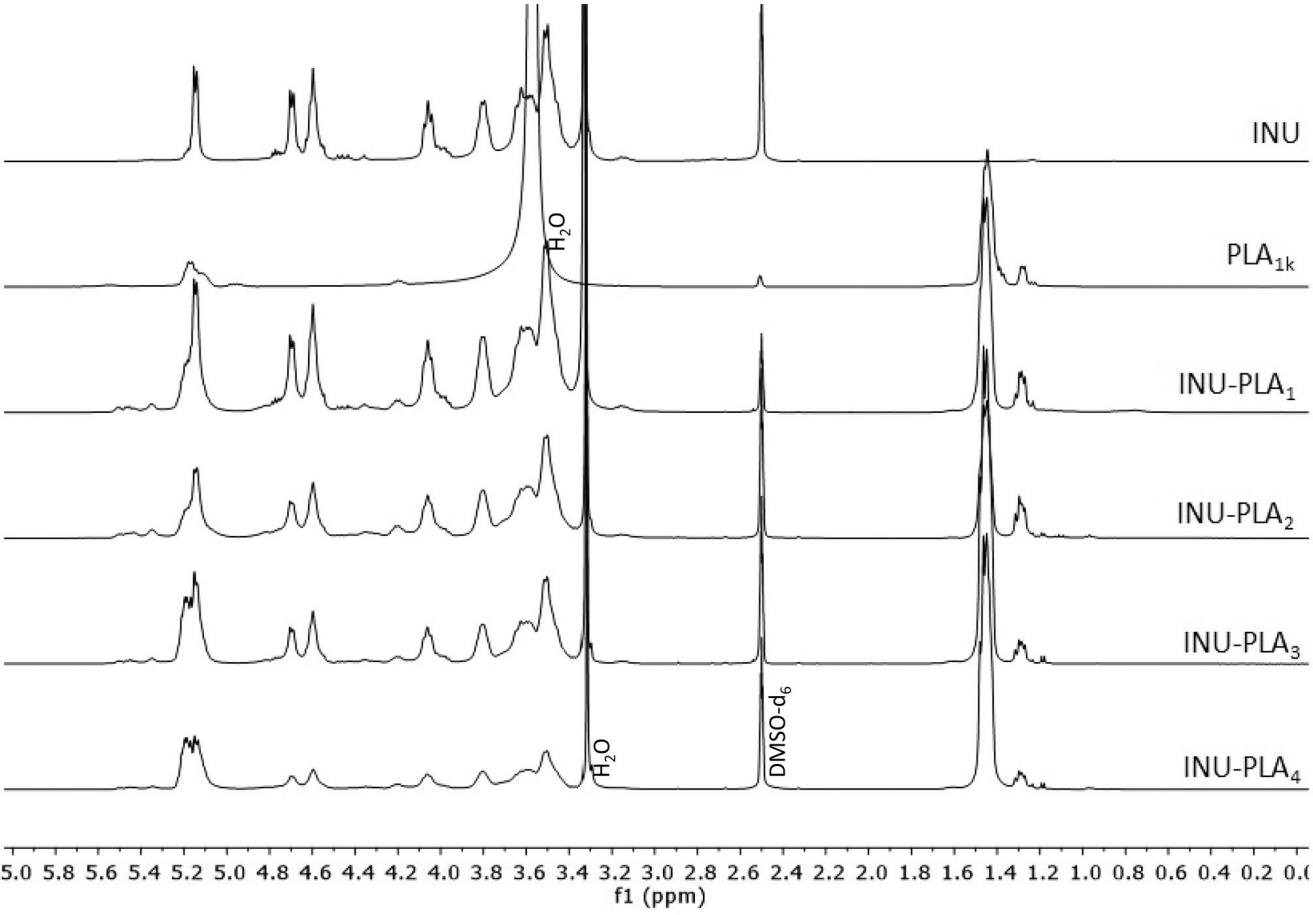
^1^H NMR spectra of INU, PLA e INU-PLA 1–4

Products obtained after purification were analyzed by mono- and bidimensional ^1^H, ^13^C NMR analysis in DMSO-*d*_*6*_. ^1^H NMR analysis spectra, reported in Fig. 2, showed the coexistence of the characteristic shifts of both INU and PLA and allowed to calculate the molar degree of derivatization (DD_mol_%), i.e., the average mmoles of PLA conjugated every 100 mmoles of INU repeating units.

DD_mol_% was calculated by comparing the integral of the signals comprised between 1.15 and 1.78 ppm accounting for the PLA methyl groups, with the integral of the signals comprised between 3.38 and 4.14 ppm assigned to the protons of inulin repeating unit with exception of hydroxyls (7H). DD_mol_% of the obtained copolymers are reported in Table 1.

**Table 1:** Degree of derivatization (DDmol%), number average molecular weight (Mn), weight average molecular weight (Mw), and polydispersity ($\overline{\mathrm{M} }$ w/$\overline{\mathrm{M}}\mathrm{n }$) of INU-PLA_1-4_ copolymers

	**DD**_**mol**_**%**^**a**^	**Mn**^**a**^** (Da)**	$\overline{\mathbf{M} }$ **w**^**b**^** (Da)**	$\overline{\mathbf{M}}\mathbf{n }$ ^**b**^** (Da)**	$\overline{\mathbf{M} }$ **w/**$\overline{\mathbf{M}}\mathbf{n }$
INU	/	4849	8721	6828	1.3
PLA_1k_	/	878	2085	1041	2.0
PLA_5k_	/	4226	6758	5017	1.3
INU-PLA_1_	6.8 ± 1.1	6568	10,042	7717	1.3
INU-PLA_2_	9.4 ± 0.7	7048	11,805	7966	1.5
INU-PLA_3_	4.5 ± 0.2	10,082	13,246	8597	1.5
INU-PLA_4_	9.7 ± 1.0	16,655	17,185	9971	1.7

^a^Calculated by ^1^H NMR. Mn INU = 179.15 + 162.14 × PD_INU_, PD_INU_ = 28.8. Mn PLA = 72.06 × PD, PD_PLA1K_ = 12.2, PD_PLA5K_ = 58.6. Mn INU-PLA = Mn PLA × (DD_mol_%/100) × PD_INU_ + Mn INU

^b^Obtained by gel permeation chromatography (GPC). Different conditions were used for inulin derivatives and starting PLA. Please refer to supplementary material

Noteworthy, comparison of the ^1^H NMR spectra of PLA and PLA grafted INU showed that the signals for the 3 protons of the methyl group adjacent to the PLA terminal carboxylic group (HOCH(CH_3_)C(O)O-PLA-CH(CH_3_)C(O)OH) undergo a shift, from ~ 1.37 to ~ 1.3 ppm, as a result of esterification (Fig. S1).

**Fig. 3:**
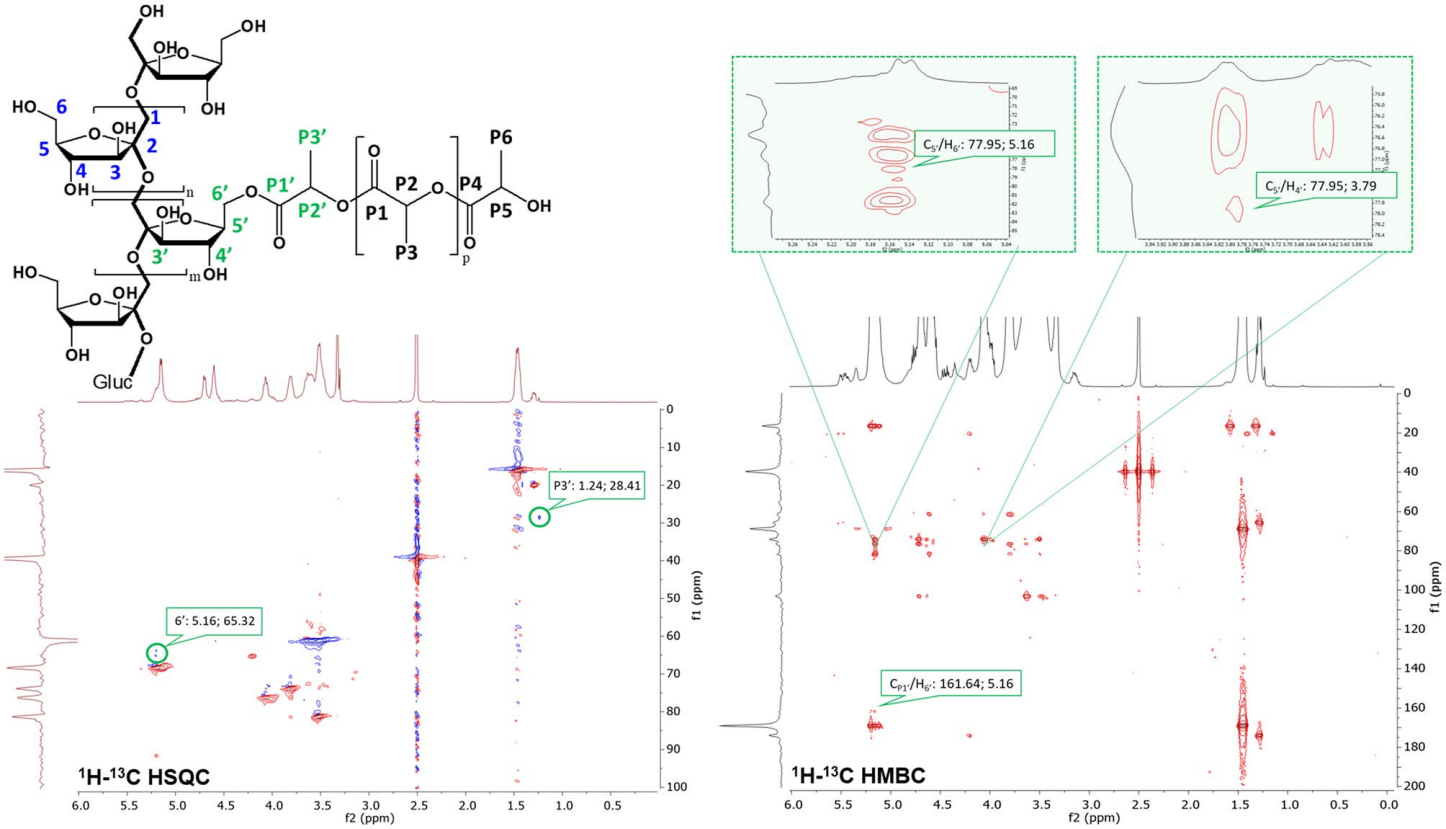
^1^H-^13^C HSQC and ^1^H-^13^C HMBC spectra of INU-PLA_1_

2D NMR spectra allowing direct or long-range correlation between ^1^H and ^13^C signals provided many useful information, especially when signals are overlapping, as often in polymer spectra, or in cases where there are characterizing heteroatoms or carbon atoms not bearing a bounded H. Figure 3 presents the ^1^H-^13^C HSQC and ^1^H-^13^C HMBC spectra of INU-PLA_1_.

^1^H-^13^C HSQC spectrum of INU-PLA showed the primary correlations from INU and the PLA side chains. In particular, the strong δC/δH correlations at 16.1/1.45, 19.9/1.29, 65.3/4.21, and 68.3/5.21 ppm were assigned to C_P3_/H_P3_, C_P6_/H_P6_, C_P5_/H_P5_, and C_P2_/H_P2_, respectively. For INU ^1^H-^13^C HSQC and full attribution of INU-PLA correlations, please refer to Fig. S2 and Table S1 in the Supplementary Materials. Moreover, new δC/δH correlations (highlighted in green in Fig. 3) at 65.3/5.16 and at 28.4/1.24 ppm appeared in the ^1^H-^13^C HSQC of INU-PLA that cannot be assigned to native polymers. Those correlations were assigned to the CH_2_ of INU-PLA in position 6′ adjacent to the substituted primary OH and to the CH_3_ of the corresponding substituted end of the PLA chain (P3′) respectively showing that PLA side chains were successfully grafted onto INU.

Observing the ^1^H-^13^C HMBC spectra of INU-PLA (Fig. 3), it is possible to appreciate specific correlations by atoms involved in the new formed bonds between INU and grafted PLA, along with the characteristic correlations between signals of the two blocks. In particular, the signal of the protons at position 6′ shows long-range correlations with the signal of the carbon P1′ δC/δH at 161.6/5.16, and the same protons were found to correlate with another carbon atom which in turn correlated with H4 and, therefore, recognized as the C5′. The two signals are highlighted in green in Fig. 3 with δC/δH at 77.9/5.16 and at 77.9/3.79 ppm, respectively. For INU ^1^H-^13^C HMBC and full attribution of INU-PLA correlations, please refer to Fig. S3 and Table S2 in the Supplementary Materials.

**Fig. 4:**
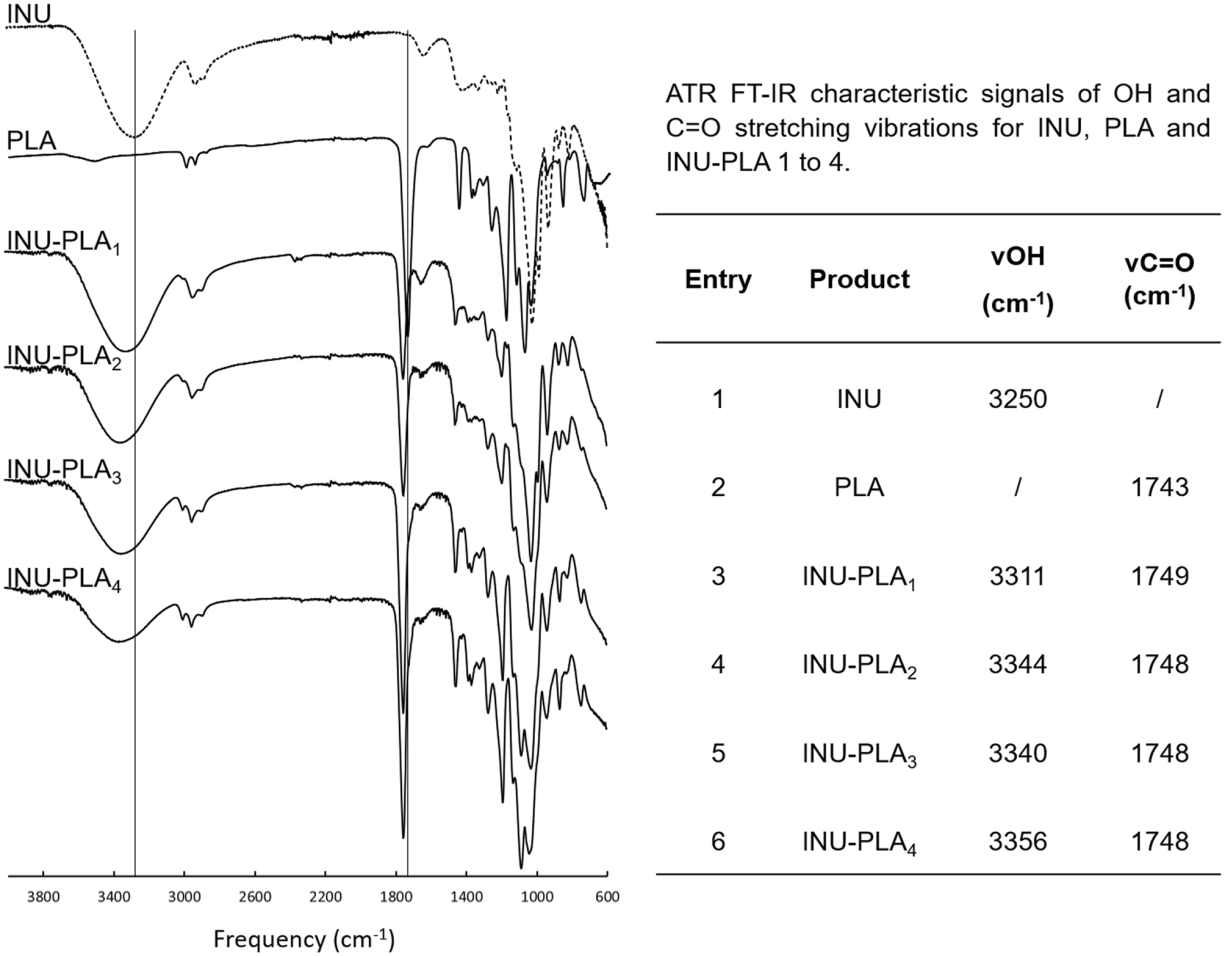
ATR FT-IR spectra of INU-PLA copolymers and precursors, PLA and INU. The inserted table show the absorption for two characteristics band: the stretching of inulin hydroxyls, around 3300 cm^−1^ and the C = O absorption band

Both raw INU and PLA and INU-PLA 1 to 4 were characterized by FT-IR spectroscopy (Fig. 4). The characteristic bands around 3300, 1027, and 934 cm^−1^ were assigned to INU backbone. The FT-IR spectrum of INU-PLA also revealed an intense absorption band from the carbonyl group (C = O) at 1748–1749 cm^−1^, confirming the presence of the PLA polymer. The latter band appeared shifted of 5 to 6 cm^−1^ if compared to the same band in raw PLA. The band for O–H stretching vibration band was shifted to a higher wave-number domain, probably due to the decreased hydrogen bonding force in INU after partial modification of its hydroxyls with PLA.

INU-PLA copolymers were also characterized by gel permeation chromatography (GPC) in DMSO at 60 °C. GPC traces are shown in Fig. S4. The elution peaks showed the complete removal of unreacted PLA homopolymer and further confirmed the successful grafting of PLA onto INU. More importantly, the products appear narrow dispersed with a PDI within 1.5, thus showing a good uniformity of the functionalization along the molecules of the carbohydrate. An exception is represented by INU-PLA_4_ for which a PDI of 1.7 was found.

Substances with amphiphilic properties have often the advantage to be soluble in a wider variety of aqueous and organic media compared to isolated blocks. This potentially improves their workability in a bigger scale and broads the solvent selection during the preparation of drug loaded nanoparticles, taking into account toxicity and polymer/drug solubility aspects. INU has a limited dispersibility in organic solvents, being DMSO almost the only one in which the oligo-fructans are freely soluble at high concentration at room temperature. Despite the high hydrophilicity, the solubility of INU in water at room temperature is poor as well, being impaired by the dense network of H-bonds between sugars.

**Fig. 5:**
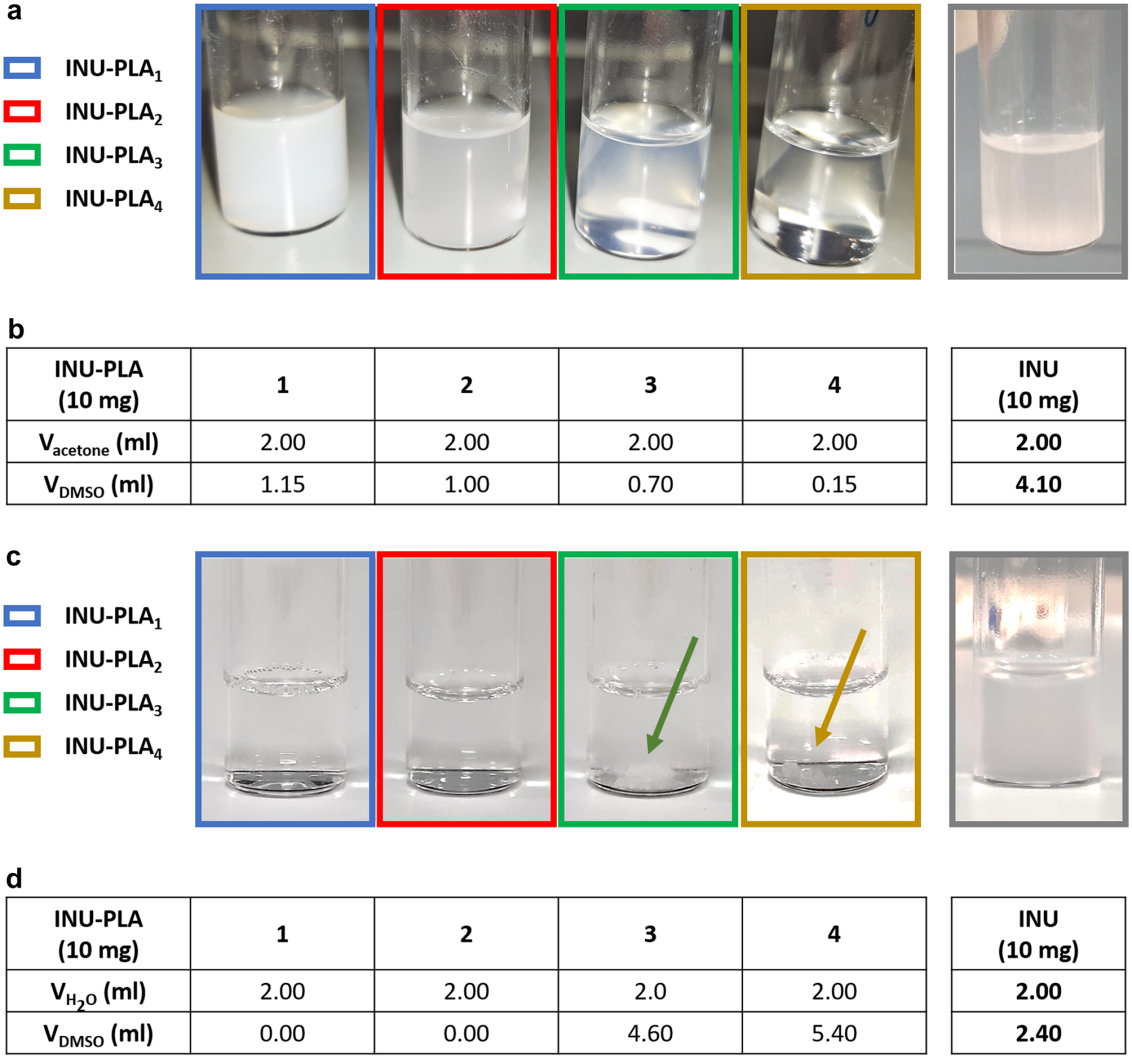
Behavior of INU-PLA derivatives in dispersion in organic solvents and in water. **a** Appearance of INU-PLA_1-4_ in acetone after the addition of 0.15 ml DMSO; **b** volumes of DMSO needed to dissolve the solid in acetone suspension; **c** appearance of INU-PLA_1-4_ in water after the addition of 0 ml DMSO; **d** volumes of DMSO needed to dissolve the solid in water suspension

The affinity of the amphiphilic INU-PLA copolymer with organic solvents compared to INU increased. This was shown by estimating the amount of DMSO needed to dissolve a suspension of INU-PLA_1-4_ in a fixed amount of acetone (a very poor solvent for INU) (Fig. 5a–b). Increasing both DD_mol_% and the molecular weight of the grafted PLA, the affinity for acetone increased, with INU-PLA_4_ needing only 0.15 ml DMSO, i.e., the 3.7% of the DMSO needed to bring INU in solution from acetone dispersion (4.1 ml). Noteworthy, grafting of PLA_1k_ drives the copolymer towards a full dispersibility in water at room temperature (Fig. 5c). By repeating the same experiment but starting from water instead of acetone, the amount of DMSO needed to solubilize the solid decreased in the order INU-PLA_4_ > INU-PLA_3_ > > INU-PLA_2_ = INU-PLA_1_ (Fig. 5d), with INU-PLA_1_ and INU-PLA_2_ no needing DMSO to be added, but gave place to a homogeneous, clear dispersion at once after water addition.

### Thermal analysis of INU-PLA copolymers

**Fig. 6:**
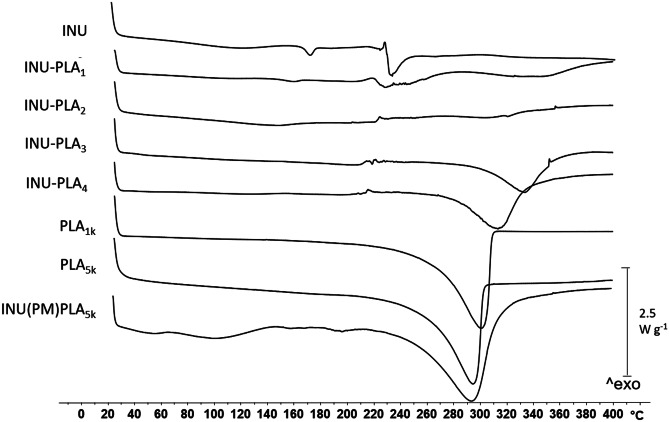
Thermograms obtained by DSC of INU-PLA_1-4_ copolymers and starting materials, INU and PLA

Thermal analysis allows correlation of thermal properties of copolymers (crystallinity, Tg, decomposition temperature) with drug release behavior, stability of formed micelles, and affinity with a drug. Thermograms of unmodified INU, INU-PLA copolymers, and PLA, obtained by DSC, are presented in Fig. 6. To remove residual solvents and erase the thermal history of the sample, results of second heat cycle were mostly taken, except for neat PLA and INU-PLA_2_, for which second-order transitions were appreciable only at higher heating rate (30 K min^−1^) and in the first step (thermograms obtained by DSC of PLA_1k_ and PLA_5k_ recorded at the first heating cycle are reported in Figs. S5 and S6).

In the current work, the inflection point instead of midpoint was chosen for the determination of the glass transition temperature (T_g_), to minimize the onset-/endset-related variation. No melting temperature (T_m_) was observed in the curve of unmodified PLA_1k_ and PLA_5k_, suggesting that the polyesters were amorphous. This was predictably ascribed to the random distribution of D- and L-chiral centers along the polymer backbone, which disturbs the regular crystalline lattice [25]. INU exhibited, instead, a T_m_ and a T_g_ of approximately 174 °C and 93 °C, respectively, revealing the semicrystalline nature of the material. For the INU-PLA copolymers, a reduced crystallinity was found, and each curve exhibited a single T_g_. Thus, modification resulted in a material with thermoplastic behavior and a reduced T_g_ to about 40–50 °C (Table 2). For details of DSC curves showing Tg of INU, PLA, and INU-PLA_1-4_, please refer to Figs. S5–S8). This indicates two possible phenomena, occurring simultaneously: first, that grafted PLA side chains expanded the distance between polysaccharidic chains and increased their degree of freedom, thus playing a plasticizer-like role (this effect was previously observed by Zhang et al. for xylan-g-PLA [26]), and second, the breaking of non-covalent interactions (hydrogen bonding) within the dangling fructose units due to functionalization of hydroxyls led to augmented mobility of polymer chains, thus decreasing the T_g_. This is substantiated by the shift of the hydroxyls’ absorption band around 3300 cm^−1^ in the ATR FT-IR spectra of INU-PLA copolymers compared to raw INU (compare Fig. 4). Changings due to the lost capacity of fructose to H-bond one another are not new in INU chemistry: it is frequently seen that after functionalization, even with hydrophobic small molecules, the dispersibility in water at room temperature increases when compared with raw INU, which is poor in water at room temperature [24].

**Table 2:** Glass transitions for INU-PLA_1-4_ copolymers and their precursors

**Sample**	**ΔCp (W **g^−1^**)**	**Onset (**°**C)**	**Endset (**°**C)**	**T**_g_ **(**°**C)**
INU	−0.18	78.25	112.77	93.07
PLA_1k_	−93.82·10^−3^	54.80	65.86	61.25
PLA_5k_	−84.32·10^−3^	53.43	63.78	58.34
INU-PLA_1_	−0.10	35.62	103.75	44.41
INU-PLA_2_	−0.1	44.17	90.47	49.91
INU-PLA_3_	−0.17	45.78	92.00	50.39
INU-PLA_4_	−0.21	49.99	95.41	54.41

Noteworthy, when PLA is grafted onto INU, the degradation of the polyester chain occurred at a higher temperature: while degradation peak was observed between 294 and 301 °C for neat PLA or PLA in the physical mixture with INU, in the case of grafted PLA, an increase up to 40 °C was observed. Data, the whole set of which is reported in Table 3, indicate the improvement in thermal stability, caused by the modification of carboxyl functional end group by grafting onto INU. An increase in the degradation temperature is compatible with the modification of the end group of a PLA chain as described earlier [27]. These results suggest that INU could interfere with one of the chemical processes involved in the thermal degradation of PLA, such as intra- or intermolecular transesterification, cis-elimination, and radical and concerted nonradical reactions [28].

**Table 3:** Degradation temperatures measured by DSC for INU-PLA copolymers, compared with starting materials

	^a^INU(PM) PLA_5k_	PLA_1k_	PLA_5k_	INU-PLA_1_	INU-PLA_2_	INU-PLA_3_	INU-PLA_4_
Integral (mJ)	−1042.42	−3061.57	−2089.37	−326.70	−474.73	−990.43	−1353.17
Onset (°C)	254.95	266.83	261.13	300.08	276.39	309.00	291.87
Peak (°C)	295.69	300.41	294.52	348.08	320.67	333.47	316.01
Endset (°C)	313.52	308.47	302.40	378.9	353.29	353.04	332.41

^a^Physical mixture between INU and PLA_5k_

### The compartmentalization of new amphiphiles: preparation and characterization of INU-PLA-based nanosystems

Once the amphiphilic copolymers were obtained and characterized, the next step was to study their ability to self-assemble into nanostructured systems in aqueous environment and to load doxorubicin (Doxo) as model anticancer drug.

Firstly, a comparison between the ^1^H NMR spectra of INU-PLA in D_2_O and in DMSO-d_6_ was carried out. Fig. S9 shows the spectra of INU-PLA_2_ in both solvents. The analysis of both spectra revealed that the signals which belong to -CH_3_ and -CH protons of PLA grafted chains have a lower resolution in D_2_O in comparison with the corresponding signals detected in DMSO-D6. Indeed, the ratio between the integrals of the signals of INU backbone and the signal of PLA methyls reduced from 0.45 in DMSO-D6 to 0.19 in D_2_O. Such an attenuation of signals together with disappearance of the splitting in ^1^H NMR spectra indicates that the hydrophobic part of the graft copolymer has a lower mobility. The reduced degree of freedom is suggestive of the PLA chains being in a confined structure such as the inner core of a water dispersed colloid [29, 30].

**Fig. 7:**
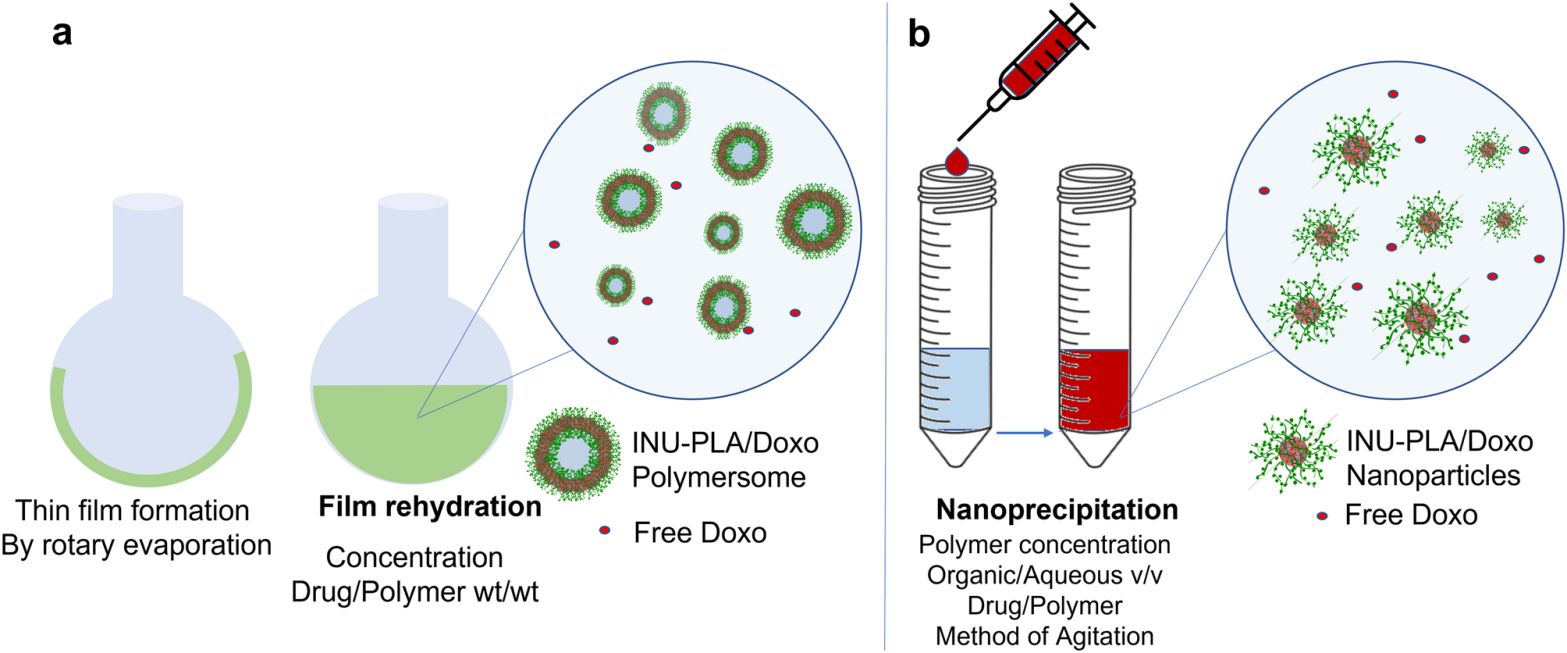
Schematic representation of the different steps involved in the film rehydration method (**a**) and the nanoprecipitation method (**b**) applied to INU-PLA. Both the procedures involve also dialysis against pure water, filtration, and freeze-drying (not showed)

Among different methods used to produce nanoparticles from amphiphilic copolymers, two of them were selected to explore the behavior of INU-PLA_1-4_ copolymers in water and to optimize the preparation of drug-loaded nanoparticles: the thin film rehydration method and the nanoprecipitation method. The methods are schematically represented in Fig. 7.

**Fig. 8:**
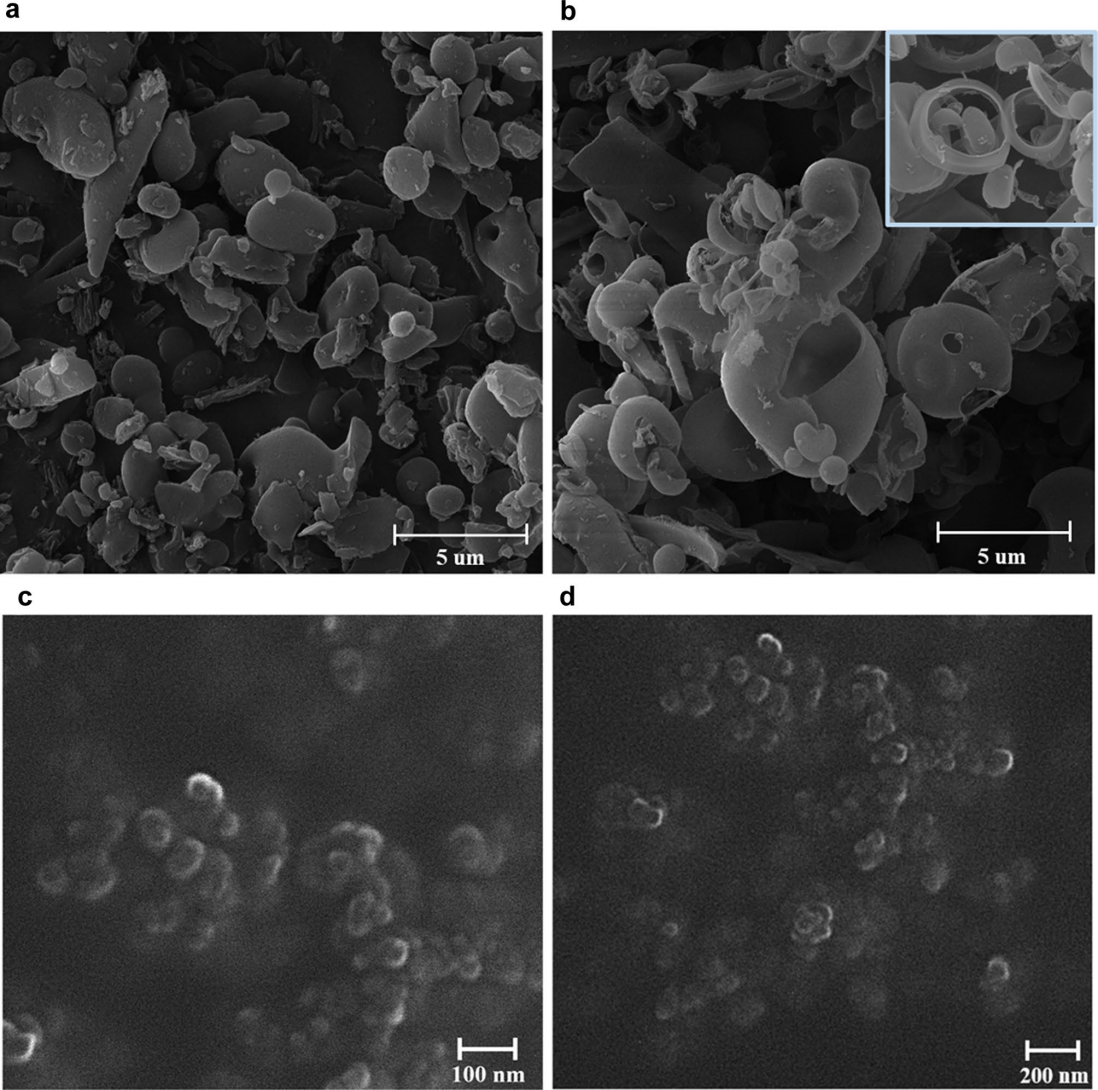
Representative SEM micrographs of the freeze-dried powder obtained from INU-PLA_2_ (**a**) and INU-PLA_4_ (**b**) by the film rehydration method. Representative SEM micrographs of the freeze-dried powder obtained from INU-PLA_2_ (**c**) (average diameter = 70.72 ± 18) and from INU-PLA_4_ (**d**) (average diameter = 73.79 ± 17) by the nanoprecipitation method. Average diameter measured by ImageJ on micrograph analysis

In the thin film rehydration method, the addition of water to the casted thin film of INU-PLA_3_ and INU-PLA_4_ caused the detachment of large film fragments, and only 10 to 30% of the starting weight was recovered after prolonged sonication, filtration, and freeze-drying. On the contrary, INU-PLA_1_ and INU-PLA_2_ were readily dispersible giving a clear phase, and the yields of the obtained solids were calculated to be over 85%. With the aim to characterize the morphology and dimensions of the obtained systems, freeze-dried samples were visualized by SEM and analyzed by DLS after dispersion in double distilled water and filtration over 0.45-µm syringe filters. The two analyses gave different results: while DLS of filtered samples revealed homogeneous population of particles in the range 160–220 nm with a zeta potential in the range of − 18 to − 31 mV in all the cases, and on the other side, at the SE microscope, a completely different scenario emerged. As can be seen in Fig. 8a and b, the solid appeared mostly formed by nonuniform microparticulate, showing a very high heterogeneity of samples produced by the thin film formation-rehydration method. Surprisingly, rehydration of INU-PLA_4_ from the film gave rise to stable giant polymersomes, a fraction of them still integer and not collapsed after freeze-drying (Figs. 8b and S8). Detailed information on the size of systems prepared by both the thin-film hydration and nanoprecipitation methods at different stages of particle preparation are reported in Table S3.

It is already known for block copolymers that they can self-assemble from spheres to polymersomes by control over the amphiphilicity. The key to this phenomenon is the hydrophobic/hydrophilic ratio. Here, the molecular weight ratio of hydrophobic to hydrophilic segments (fH/H) for INU-PLA_1-4_ and hydrophilic-lipophilic balance (HLB) is reported in Table 4. The lower the hydrophilicity, the lower is the interfacial curvature of the copolymer, which pushes the assembly towards vesicles instead of micelles or nanoparticles [28]. Therefore, it is not particularly surprising that in our case (even if INU-PLAs are graft and not block copolymers), once the right f_H/H_ is reached, a change in the morphology of the obtained system occurred. This was not observable by changing the method to nanoprecipitation. Indeed, in this second case, SEM analysis showed in all cases population of small nanoparticles with diameter around 70 nm (Fig. 8c and d). Moreover, the obtained yields were high, and after DLS analysis, we found results in accordance with the two techniques.

**Table 4:** The molecular weight ratio of hydrophobic to hydrophilic segments (fH/H) and hydrophilic-lipophilic balance (HLB) for INU-PLA_1-4_ (f_H/H_) for INU-PLA_1-4_ and PEG-PLA

**Copolymer**	**Equivalent linear formula**	**fH/H**	^1^**HLB**
INU-PLA_1_	INU_4849_-PLA_1719_	0.4	14.8
INU-PLA_2_	INU_4849_-PLA_2377_	0.5	13.4
INU-PLA_3_	INU_4849_-PLA_5477_	1.1	9.4
INU-PLA_4_	INU_4849_-PLA_11807_	2.4	5.8
PEG-PLA	PEG_2000_-PLA_2200_	1.1	9.5

^1^Calculated by the general expression given by Griffin [31] HLB = 20*(Mh/M), where Mh is the molar mass of the hydrophilic portion and M is the molar mass of the whole macromolecule

The above results indicate that in conditions where the formation of lyotropic phases (such as a lamellar phase) occurs after hydration (such as in the case of film rehydration method), the hydrophobic PLA segments in INU-PLA interact and freeze in a local minimum of energy from where bending and fitting in the interior of a core/shell nanoparticle is highly disfavored, which is why the copolymer tends to form bigger particles and vesicles, such as polymersomes in case of INU-PLA_4_ which has the highest f_H/H_.

On the contrary, during nanoprecipitation, a process that allows the formation of the nanosystem via nucleation in dependence on the balance between the assembled state and unimers in dispersion, the final architecture is not matured from an intermediate state, but it is “trapped” in the nucleus shape, which is maintained during growing eventually [28]. Information on the size of systems prepared by both the thin-film hydration and nanoprecipitation methods at different stages of particle preparation is reported in Table S3.

HLB values determined for INU-PLA_1-4_ follow in the range 6–15. Some ester derivatives of inulin [32–34] with HLB values comprised between 7 and 17 have been found to be good emulsifiers, potentially employable in pharmaceutical as well as in other technological field such as cosmetics, food chemistry, detergents, and bioplasticizers.

### INU-PLA-based nanosystems as drug carrier

Following the above preliminary results, we chose to use nanoprecipitation method for further investigation on drug loading ability of the nanosystem. Thus, we explored the effect of the method parameters (polymer concentration, theoretical drug loading, and homogenization method) on the size distribution and actual drug loading (DL%).

**Fig. 9:**
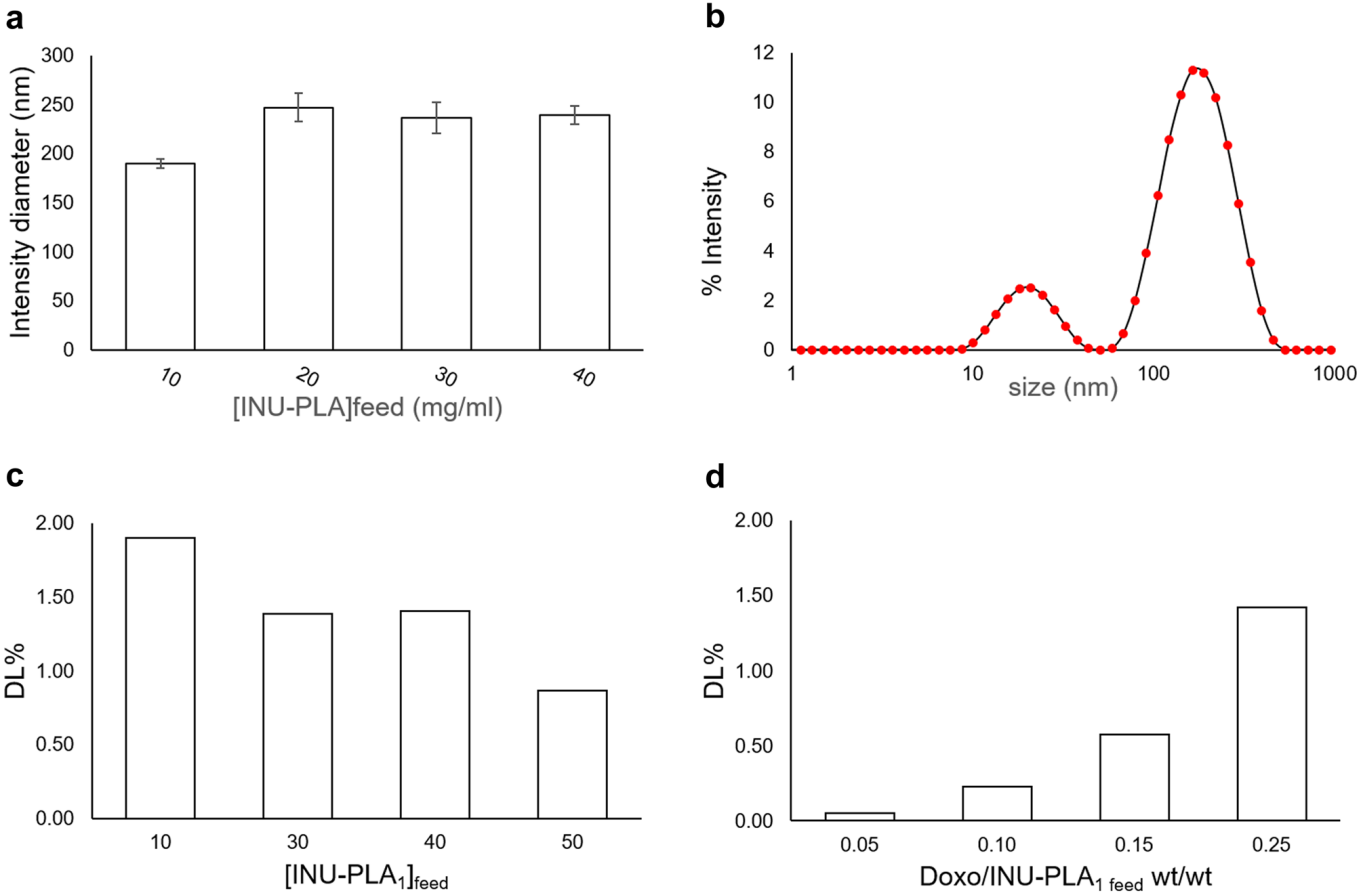
Characteristics of nanosystems obtained by INU-PLA_1_. **a** Comparison of the size of the empty nanosystems obtained by nanoprecipitation at different polymer concentration in the feed solution ([INU-PLA_1_] feed); **b** intensity vs size plot for the system obtained by nanoprecipitation using a [INU-PLA_1_]feed of 10 mg/ml. **c** Ability of INU_PLA1-based systems to incorporate doxorubicin as the polymer concentration and amount of drug **d** changes

In a first set of experiments, we used INU-PLA_1_ and the homogenization by ultra turrax at 0 °C (to avoid excessive foaming and to contrast heat production due to metallic part rotation). The polymer concentration in the feed solution ([INU-PLA_1_]feed) was changed in the range 10–50 mg/ml, and the hydrodynamic diameter was followed by DLS measurements of samples redispersed in water after freeze-drying. Changing [INU-PLA_1_]feed appeared to have little or no influence on the hydrodynamic diameter (Fig. 9a). The only exception occurred when the concentration was below 20 mg/ml: in that case, the size statistically decreased by about 50 nm, and a bimodal distribution characterized the intensity vs size plot, with the appearance of a narrow population around 20 nm (Fig. 9b).

Under the same conditions, Doxo was added to the feed polymer solutions at a Doxo/INU-PLA_1_ of 20% wt/wt. Although the effect of Doxo encapsulation on particle diameter appeared negligible in the range 20–40 mg/ml, the repercussion of polymer concentration on drug loading seems not to be trivial, with a surprising decrease in DL% with increasing [INU-PLA_1_]feed from 1.90 to 0.87% (Fig. 9c). By keeping fixed the [INU-PLA_1_]feed (40 mg/mL), and increasing the Doxo/INU-PLA_1_, a progressive increase in the amount of drug entrapped was found. In particular, results show that an increase in Doxo/INU-PLA_1 feed_ from 5 to 20% by weight led to an increase in DL% from 0.1 to 1.4% (Fig. 9d). Clearly, higher theoretical loadings correspond to a greater number of drug molecules available to be trapped inside particles, leading to the greater DL% observed.

**Fig. 10:**
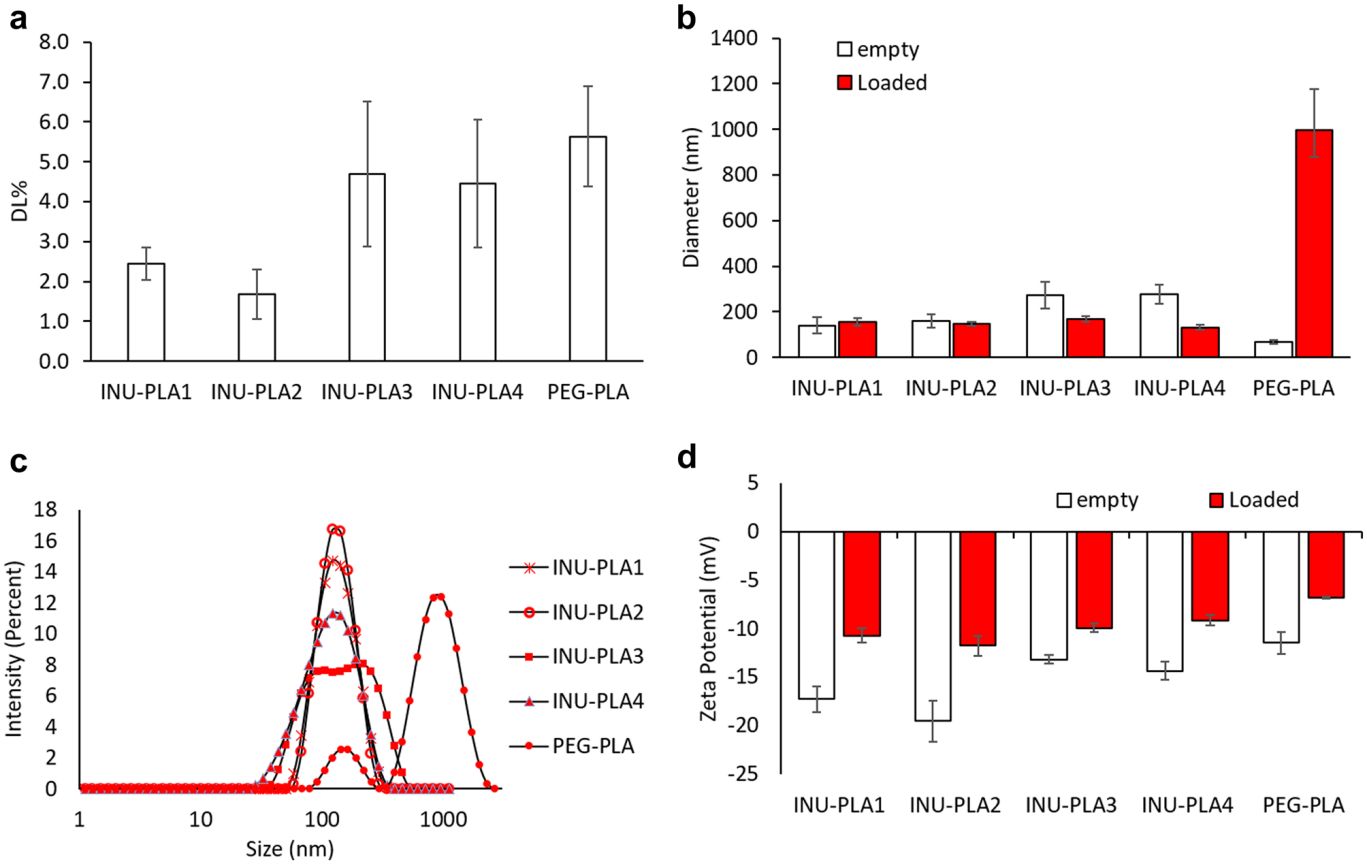
Drug loading (**a**), size (**b**–**c**), and zeta potential (**d**) of INU-PLA_1-4_ nanosystems. Panel (**c**) reports the size distributions of loaded systems obtained by DLS measurements of samples dispersed in water after freeze-drying

Using vortexing at room temperature as a method of homogenization, a sharp increase in drug loading was achieved up to 5%. The reason why switching from ultra turrax to vortex cause such an increase in the ability of the system to entrap the drug could be ascribed to the different rate Doxo is able to diffuse in the water phase while the nucleation occurs (higher for ultra turrax), as well to the higher temperature during vortexing. The data also showed that INU-PLA is able to load Doxo to the same extent as PEG-PLA. In particular, for INU-PLA_3_ and PEG-PLA having the same f_H/H_ of 1.1, we found a DL% of 4.7 ± 1.8% and 5.6 ± 1.3%, respectively. Results are summarized in Fig. 10a.

The dispersion of the drug in the hydrophobic core alters to some extent the overall hydrophilic-hydrophobic balance [35, 36]. The effect increases with increasing of the drug loading, and it is reflected on the size difference between empty and loaded nanosystems. For systems obtained starting from INU-PLA_1_ and INU-PLA_2_, in which the amount of Doxo entrapped is averagely 24.4 ± 4.2 µg/mg and 20.3 ± 2.7 µg/mg, respectively, the difference between empty and loaded particles is not statistically relevant. Going to INU-PLA_3_ and INU-PLA_4_, which were able to entrap almost double the amount of Doxo compared to the analogues 1 and 2 (47.0 ± 18.2 and 44.6 ± 16.0 µg/mg, respectively), a more pronounced effect on the drug dispersed in the core was observed as a difference in size between empty and loaded systems (Fig. 10b).

While the dispersed drug has stabilizing activity in the case of INU-PLA graft construct assembly, the contrary occurred for PEG-PLA, in which case the colloidal stability was almost lost after drug incorporation and freeze-drying (Fig. 10c). The better re-dispersibility of INU-PLA, without the need of a cryoprotectant, represents an advantage in the processability and translatability of the systems. It is not surprising that INU gave such an effect, since it is already known its ability to act as a cryo- and lyoprotectant [37].

Differential scanning calorimetry (DSC) showed that Doxo is in the nanosystems in a non-crystalline form, as no melting transition characteristic of Doxo could be measured, suggesting that the drug is molecularly dispersed in the systems (Fig. S10). Moreover, we cannot exclude the drug that is partially adsorbed on the outer hydrophilic shell, being the zeta potential after dialysis purification and freeze-drying slightly less negative for Doxo loaded INU-PLA nanoparticles compared to empty ones (Fig. 10d).

Surprisingly, when the INU-PLA copolymer was used in the formation of particle systems (INU-PLA/Doxo), a double second-order transition corresponding to two different Tg were observed: one at about 50 and one at about 70 °C (Fig. S11). The phenomenon, although not entirely clear, is suggestive of a segregation between the two polymer components and has been observed as a consequence of nanoparticle confinement in core–shell systems [38, 39].

**Fig. 11:**
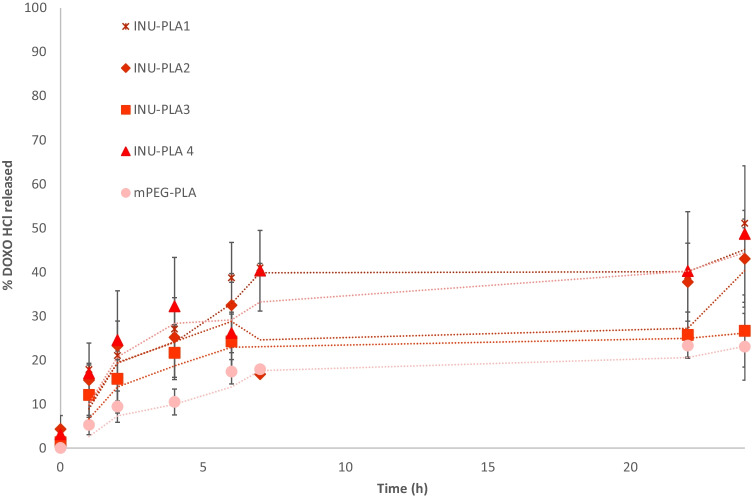
Drug release from INU-PLA/Doxo and PEG-PLA/Doxo nanoparticles. Results are reported as mean ± SD from three different experiments

Finally, in vitro Doxo release was evaluated in TRIS buffer (5 mM, pH = 7.4) at 37 °C. During experiments aiming to estimate the amount of free Doxo diffused with time, only around 75% of drug was released after 24 h, and no further release was detected by UV–VIS analysis (Fig. S12). This behavior was due to the degradation of Doxo as well as its retention after membrane adsorption. As a consequence of its poor stability, once released from the nanoparticles, Doxo was indeed partially degraded in the release medium, thus making analysis impossible for the longer time points. Within 24 h, it is possible to observe a different release kinetic for INU-PLA_1-4_ (Fig. 11).

In particular, increasing the f_H/H_, the Doxo release slowed down with the maximum amount of Doxo released after 24 h being that of INU-PLA_1_/Doxo nanoparticles (56 ± 6% of the loaded Doxo). An exception is represented by INU-PLA_4_-based nanoparticles, showing a lower repeatability and a big deviation on average results. This could be due to the higher heterogeneity of the starting copolymer in terms of molecular weight distribution (PDI = 1.7, cfr Table 2) leading to nanoparticles with a higher heterogeneity in the composition.

Drug release is apparently in contrast with DSC data. For a polymeric micellar type, a lower Tg should correspond to a lower CMC, a higher core dynamicity, and an easier exchange between the core and the external medium, with a consequent faster drug release. Therefore, it is evident that the new INU-based materials are able to self-assemble in a frozen state (not micellar) with a very slow rate of exchange (kinetically frozen aggregates) that can be slowed down by the length of the hydrophobic PLA core. Block INU-PLA copolymers with the same H/H ratios would all normally bring to a micellar system.

## Conclusion

A first family of Inulin-g-poly-D,L-Lactide amphiphilic copolymers (INU-PLAs) was synthesized and characterized. Composition differences between the graft INU-PLA_1-4_ copolymers seem to reflect on the physicochemical characteristic (i.e., solubility, affinity for water, and organic solvents, Tg) as well as in the behavior as colloids able to encapsulate and release an anticancer drug. By functionalization of INU with PLA_1K_, water dispersible polymer is obtained, while grafting of PLA_5k_ leads to water insoluble amphiphiles. From these, namely INU-PLA_1-2_ and INU-PLA_2-4_, it was possible to obtain nanosized particles with similar characteristics in terms of recovered amount after processing, size distribution, and zeta potential. Dependently from the method used for their preparation, nanoprecipitation, or film rehydration method, the systems were able to assemble in microparticle/micro-polymersome or nanoaggregate forms. All the grafted copolymers are able to entrap a significative amount of doxorubicin (Doxo) in a stable colloid and to release it with a kinetic modulable with the core/corona ratio. The results encourage us to continue exploring the potential of INU-PLAs as a new renewable material for biomedical application. In particular, further studies are needed to address the performance of INU-PLA nanoparticles in vitro and in vivo and to compare it with that of conventional nanoscale drug delivery systems.

## Experimental part

### Materials

Inulin from dahlia tubers (INU, Mw ≈ 5000 Da), doxorubicin hydrochloride (Doxo HCl), 6-Azido-6-deoxy-D-galactose (GalN_3_), triethylamine (NEt_3_), carbonyl diimidazole (CDI), and diethyl ether (EtOEt) were purchased from Merck. Poly(D,L-lactide) acid endcap (PDLLA, Mn: 1,000–5,000), Poly(D,L-lactide) acid endcap (PDLLA, Mn: 5,000–10,000), and Methoxy poly(ethylene glycol)-b-poly(D,L-lactide) (mPEG-P-D,L-LA, Mn: 2,000:2,200), were obtained by PolySciTech (Division of Akina, West Lafayette, IN). INU was dried at 70 °C overnight in an oven and cooled down under vacuum over P_2_O_5_ prior to be dissolved for reactions. All the solvents were used as received. Dialyses were carried out in RC Spectrapor dialysis tubes (Repligen, MA, USA).

NMR spectra were recorded in DMSO-d_6_ using a Bruker Avance III spectrometer operating at 400 MHz. FT-IR spectra were recorded on the solid samples using a Frontier FT-IR spectrometer (Perkin Helmer) equipped with ATR single reflection sampling module.

### Gel permeation chromatography (GPC)

The GPC system used consisted of a pump (LC-20AD, Shimadzu, Kyoto, Japan), a degassing unit (DGU-20A3R, Shimadzu, Kyoto, Japan), a forced air oven, two *in serie* GPC column packed with styrene–divinylbenzene copolymer gel (500 Å and 10^3^ Å fixed pore columns) (Phenogel, from Phenomenex srl, Italy) preceded by a guard column and a refractive index detector (Refractive Index Detector-10A, Shimadzu, Kyoto, Japan). GPC conditions were as follows: 2.5 mg/mL as sample concentration, 100 µL of injection volume, 0.6 mL/min flow rate, and the column temperature set to 60 °C. The detector cell of RID was kept at 60 °C. The elution solvent was pure DMSO. Samples were filtered through 0.45-µm PTFE syringe filters before injection. Data acquisition was carried out using Lab Solutions Lite software (Shimadzu, Kyoto, Japan). Detailed information about processing and calibration are reported in Fig. S13.

### General procedure for the synthesis and characterization of Inulin-graft-poly-D,L-lactide (INU-PLA) copolymers

INU (100 mg, 61.67 · 10^−2^ mmol of fructose repeating units) was dissolved in 1.2 mL of a-DMF at room temperature (4 h). TEA (0.172 mL, 2 eq/mmol INU repeating units) was then added and the mixture stirred at 40 °C for few minutes. A solution of P-D,L-LA acid end-cap (0.06 or 0.12 eq/mmol INU repeating units in 3 mL of a-DMF), previously activated with 2 eq of CDI for 4 h at 40 °C, was then added, and the mixture was kept under magnetic stirring at 40 °C for 67 h. After this time, the mixture was dropped into an excess of EtOEt. The obtained solid, collected by centrifugation (ALC PK120 centrifuge, 1129 rcf, 10 min, rt), was washed with 40 mL of the same solvent and then with 35 mL of a mixture of EtOEt/Acetone 85:15 for two times. The isolated solid was then dried under vacuum. The pure product in the form of a white solid. ^1^H NMR (400 MHz, DMSO-d_6_, ppm) δ 5.54 – 5.40 (m, 1H, PLA-C***H***(CH_3_)C(O)OCH_2_-INU, 5.35 (s, 1H, ***H***O-CH(CH_3_)C(O)O-PLA), 5.25 – 5.14 (m, 1H, PLA-C***H(CH***_3_)C(O)O-PLA), 5.14 (d, J = 5.5 Hz, 1H, CH(4)OH fructose), 4.69 (d, J = 6.3 Hz, 1H, CH(3)OH fructose), 4.59 (t, J = 4.7 Hz, 1H, CH_2_(6)O***H***fructose), 4.20 (dd, J = 13.0, 6.1 Hz, 1H, HO-CH (CH_3_)C(O)O-PLA), 4.06 (t, J = 7.2 Hz, 1H, C***H***(4)OH fructose), 3.80 (d, J = 4.7 Hz, 1H, C***H***(3)OH fructose), 3.74 – 3.38 (m, 5H, C***H***_***2***_(6)C***H***(5) + C***H***_***2***_(1) INU), 1.46 (q, J = 5.5 Hz, 3H, PLA-CH(C***H***_***3***_)C(O)O-PLA), 1.35 – 1.20 (m, 6H, HOCH(***CH***_***3***_)C(O)O-PLA-CH(***CH***_***3***_)C(O)O-INU).

FT-IR (ATR, cm^−1^) 3312, 2940, 3887, 1749, 1647, 1456, 1381, 1327, 1260, 1192, 1129, 1082, 1027, 988, 934, 871, 819.

### General procedure for the preparation of doxorubicin loaded INU-PLA nanoparticles by nanoprecipitation

Doxo-HCl was dissolved in DMSO and neutralized with 4 mol excess of TEA. The Doxo solution was mixed with the polymer solution in DMSO. The mixture was added dropwise to double distilled water and homogenized by using an ultra turrax (T 25, Ika) for 3 min at 20,000 rpm. The tube was immersed in an ice bath to keep the temperature between 0 and 2 °C. The obtained nanoparticles were purified by dialysis for 24 h against 1 L double distilled water using Spectra Por Dialysis Tubing with a molecular weight cut-off of 1000 Da. The dispersion was collected, sonicated for 10 min, filtrated by 5-µm pore syringe filter, frozen by immersion in a mixture of CO_2_ (s)/2-propanol and freeze-dried from water. Empty micelles were prepared by using the above reported procedure in the absence of Doxo and triethylamine. Alternatively, vortexing for few seconds during nanoprecipitation was used as homogenization method. The rest of the procedure remains the same as described above.

### General procedure for the preparation of doxorubicin loaded INU-PLA nanoparticles by film rehydration method

Doxo-HCl was dissolved in 1 ml a-DMF together with INU-PLA in a 50 ml round bottom flask and neutralized with 4 mol excess of TEA. The solution was dried under reduced pressure in a rotary evaporator at 30 °C, until a thin dry film is formed. Ten milliliters of water were slowly added under stirring and the mixture sonicated for 15 min at room temperature. The obtained mixtures were purified by dialysis for 24 h against 1-L double distilled water using Spectra Por Dialysis Tubing with a molecular weight cut-off of 1000 Da. The dispersion was collected, sonicated for 10 min, filtrated by 5-µm pore syringe filter, frozen by immersion in a mixture of CO_2_ (s)/2-propanol and freeze-dried from water. Empty micelles were prepared by using the above reported procedure in the absence of Doxo and triethylamine.

### Determination of doxorubicin loaded into INU-PLA nanoparticles

The amount of Doxo loaded into INU-PLA nanoparticles was measured by UV spectroscopy. Samples were prepared by dissolving exactly weighted amounts of freeze-dried INU-PLA/Doxo in DMSO and after dilution 1:1 with phosphate buffer (PBS) 0.1 M pH 7.5, measuring the absorbance of Doxo at 500 nm (Doxo, INU-PLA/Doxo, and INU-PLA representative UV–Vis spectra recorded between 200 and 600 nm are reported in Fig. S14a). A calibration curve was created by measuring absorbance for serially diluted solutions of Doxo-HCl in DMSO/PBS 1:1 (concentration ranging from 2.5 to 25 µg/ml—Fig. S14b). The content of drug loaded into the micelles, Drug Loading % (DL%), was calculated by the amount of total entrapped drug divided by the total nanoparticle weight. Encapsulation efficiency (EE%) was calculated by the ratio between actual and theoretical DL%.

### Size, distribution, and ζ potential

Dynamic light scattering (DLS) studies were performed at 25 °C with a Malvern Zetasizer Nano ZS instrument fitted with a 532-nm laser at a fixed scattering angle of 173° using the Zetasizer Software 8.01.4906. The intensity-average hydrodynamic diameter (nm) and polydispersity index (PDI) were obtained by cumulative analysis of the correlation function. Zeta potential measurements were performed by aqueous electrophoresis measurements, recorded at 25 °C using the same apparatus. The zeta potential values (mV) were calculated from the electrophoretic mobility using the Smoluchowski relationship. Samples were dispersed in double distilled water at a concentration of 1 mg/ml and sonicated for 60 s before measurement.

### Morphology measurements

The morphology of representative formulations was studied by scanning electron microscopy (SEM) (Tescan Solaris, Tescan Orsay Holding, Czech Republic). Analysis was conducted at 20 keV. Freeze-dried samples were dispersed on a carbon-coated aluminum stub and sputter coated with gold.

### Drug release studies

In vitro drug release study was carried out by dialysis method. An exactly weighted amount of freeze-dried Doxo loaded nanoparticle (which contained 200 µg of Doxo based on drug loading) was dispersed in 1 ml of TRIS buffer (5 mM, pH 7.4) and pipetted into a dialysis tubing (3.5 KDa MWCO, regenerated cellulose). The device was then immersed in 40 ml of release medium, with a thermostatic control at 37 °C and under stirring) 120 rpm. At scheduled time intervals, 1 ml of the medium was withdrew from the acceptor compartment and replaced with an equivalent amount of fresh TRIS buffer.

The amount of drug released was estimated by spectrophotometry measurement at 485 nm. Each experiment was carried out in triplicate, and average values of cumulative drug released were plotted.

### Differential scanning calorimetry

All measurements were made in non-hermetic aluminum crucible (40ul) in a Mettler Toledo 822e instrument. The sample amount was exactly weighed around 5 mg. Differential scanning calorimetry (DSC) curves were obtained by heating from 25 to 125 °C with rate of 30 °C/min, then cooling to 25 °C with a cooling rate of 30 °C/min, and heating again to 400 °C with rate of 10 °C/min. Experiments with only one cycle 25–400 °C with a heating rate of 10 °C/min were also carried out. During measurements, the sample cell was purged with nitrogen at a flow rate between 60 and 70 ml/min.

### Statistical analysis

The results are expressed as mean ± SD, and the statistical significance of all the results was determined by the Student’s t test. *p* < 0.05 was considered to be statistically significant.

## Supplementary Information

Below is the link to the electronic supplementary material.

Supplementary file1 (DOCX 2475 KB)

## Data Availability

Authors can confirm that all relevant data are included in the article and/or its supplementary information files. Further source data for figures are available from the corresponding author upon reasonable request.
