# Supplementary material for: Inulin-g-poly-D,L-lactide, a sustainable amphiphilic copolymer for nano-therapeutics
Source: Drug Deliv Transl Res. 2022 Feb 22;12(8):1974–90. doi: 10.1007/s13346-022-01135-4 (PMC9242920; doi:10.1007/s13346-022-01135-4)
Supplement: Supplementary file 1 — Supplementary file1 (DOCX 2475 KB) [file 13346_2022_1135_MOESM1_ESM.docx]

**Supplementary material**

Inulin-g-poly-D,L-lactide, a Sustainable Copolymer for Nano-therapeutics

Authors: Carla Sardo; Teresa Mencherini; Carmela Tommasino; Tiziana Esposito; Paola Russo; Pasquale Del Gaudio; Rita Patrizia Aquino*.


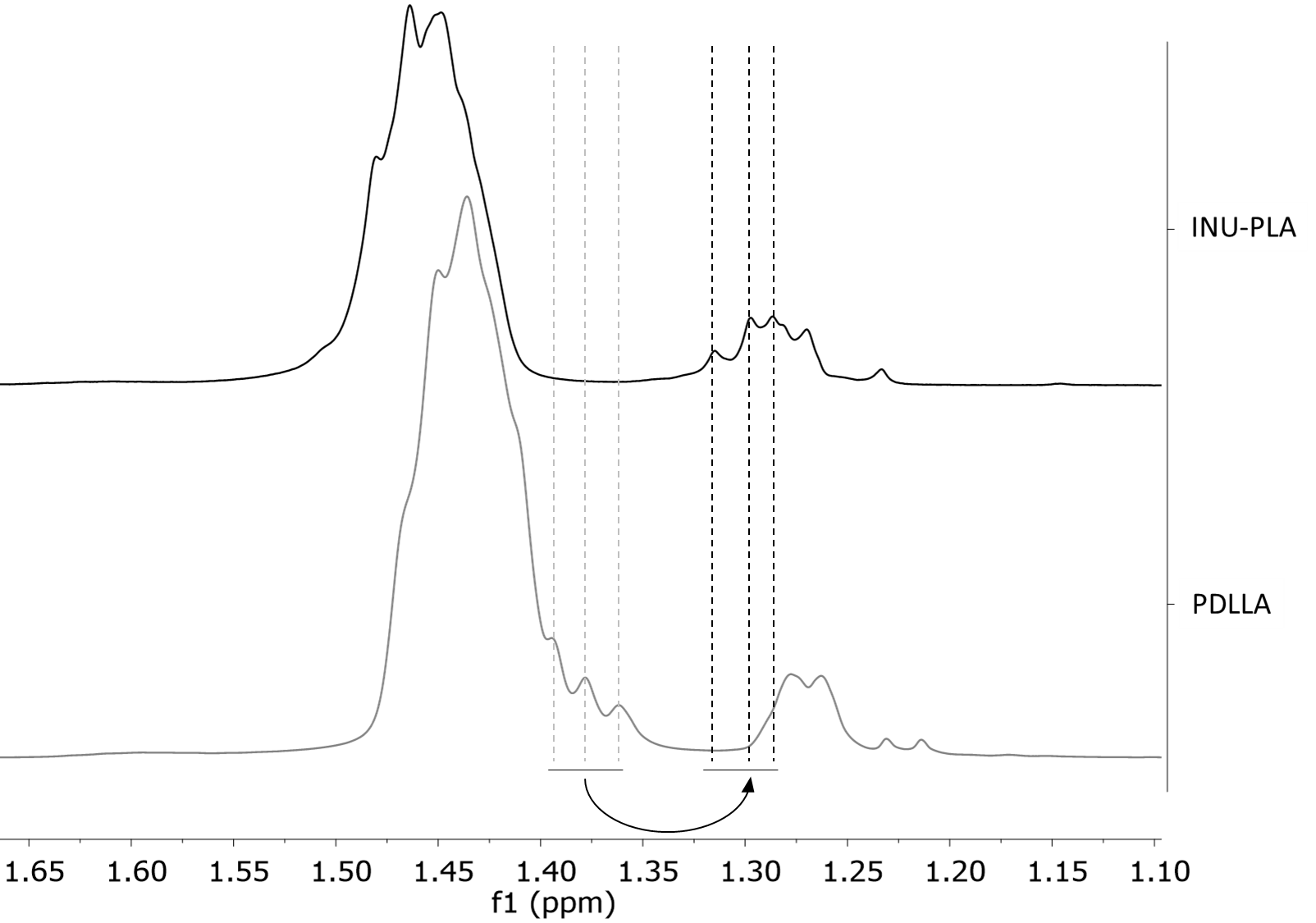


**Figure S1.** Detail of the -CH_3_ region of the 1H NMR spectra for INU-PLA and PDLLA.


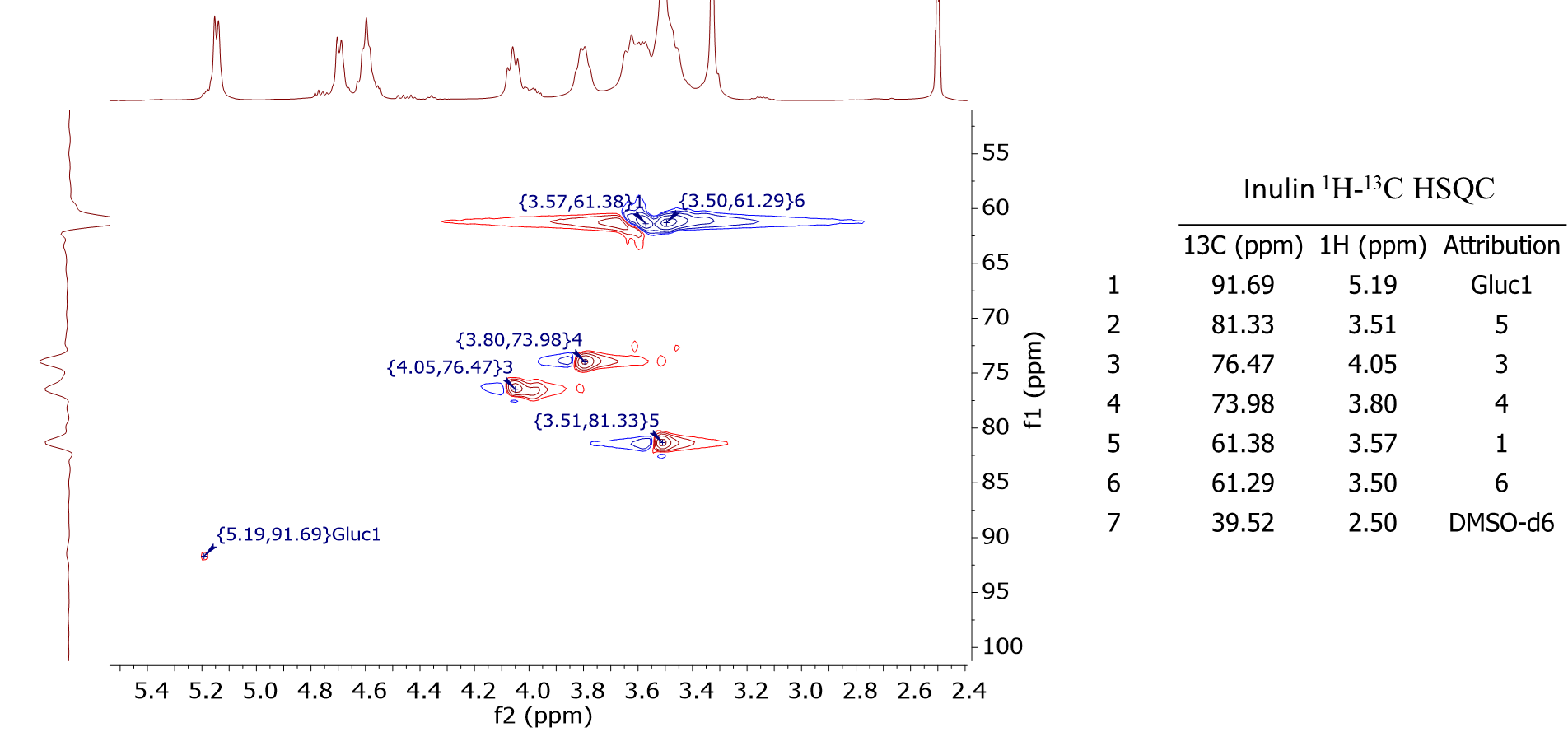


**Figure S2**. ^1^H-^13^C HSQC spectrum of INU. Recorded in DMSO-d6.

| **Table S1.** HSQC chemical shifts data of INU-PLA_1_ | | | |
| --- | --- | --- | --- |
|  | Chemical Shift | | Attribution |
|  | ^13^C (ppm) | ^1^H (ppm) |  |
| 1 | 91.70 | 5.20 | Gluc1 |
| 2 | 81.18 | 3.53 | 5 |
| 3 | 76.26 | 4.06 | 3 |
| 4 | 76.00 | 3.81 | n.a. |
| 5 | 73.77 | 3.81 | 4 |
| 6 | 68.33 | 5.21 | P2 |
| 7 | 65.32 | 5.16 | 6' |
| 8 | 65.26 | 4.21 | P5 |
| 9 | 65.20 | 5.21 | n.a. |
| 10 | 63.83 | 5.20 | n.a. |
| 11 | 61.19 | 3.59 | 6 |
| 12 | 60.92 | 3.52 | 1 |
| 13 | 28.41 | 1.24 | P3' |
| 14 | 19.91 | 1.29 | P6 |
| 15 | 16.06 | 1.45 | P3 |

**Figure S3**. ^1^H-^13^C HMBC spectrum of INU. Recorded in DMSO-d6.

| **Table S2.** HMBC chemical shifts data of INU-PLA_1_ | | | |
| --- | --- | --- | --- |
|  | Chemical Shift | | Attribution |
|  | ^13^C (ppm) | ^1^H (ppm) |  |
| 1 | 174.10 | 4.20 | HP5/ CP4 |
| 2 | 173.93 | 1.28 | CP4/ HP6 |
| 3 | 169.12 | 5.20 | CP1/ HP2 |
| 4 | 169.04 | 5.16 | CP1/H6’ |
| 5 | 169.00 | 5.12 | CP1/ HP2 |
| 6 | 168.99 | 1.46 | CP1/ HP3 |
| 7 | 161.64 | 5.16 | CP1'/ H6' |
| 8 | 103.33 | 4.71 | C2/ OH3 |
| 9 | 103.12 | 3.49 | C2/ H1 |
| 10 | 103.10 | 3.63 | C2/ H1 |
| 11 | 81.54 | 3.50 | C5/ H6 |
| 12 | 81.53 | 4.61 | C5/ OH6 |
| 13 | 81.52 | 5.16 | C5/ OH4 |
| 14 | 81.39 | 3.80 | C5/ H4 |
| 15 | 77.95 | 5.16 | C5'/ H6' |
| 16 | 77.95 | 3.79 | C5'/ H4 |
| 17 | 76.52 | 5.16 | C3/ OH4 |
| 18 | 76.52 | 3.63 | C3/ H1 |
| 19 | 76.50 | 3.80 | C3/ H4 |
| 20 | 76.42 | 4.72 | C3/ OH3 |
| 21 | 74.18 | 4.06 | C4/ H3 |
| 22 | 74.16 | 4.64 | C4/ OH6 |
| 23 | 74.15 | 5.16 | C4/ OH4 |
| 24 | 74.13 | 3.50 | C4/ H5 |
| 25 | 74.03 | 4.72 | C4/ OH3 |
| 26 | 68.91 | 5.04 | n.a. |
| 27 | 68.78 | 5.35 | n.a. |
| 28 | 68.69 | 5.31 | n.a. |
| 29 | 66.03 | 5.53 | n.a. |
| 30 | 65.57 | 5.48 | n.a. |
| 31 | 61.47 | 4.61 | C6/ OH6 |
| 32 | 61.34 | 3.81 | C6/ H4 |
| 33 | 61.34 | 4.07 | C1/ H3 |
| 34 | 39.52 | 2.50 | DMSO-d6 |
| 35 | 20.36 | 1.15 | CP6/ HP6 |
| 36 | 20.33 | 1.41 | CP6/ HP6 |
| 37 | 20.31 | 4.20 | CP6/ HP5 |
| 38 | 16.52 | 5.20 | n.a. |
| 39 | 16.48 | 1.32 | CP3/ HP3 |
| 40 | 16.48 | 1.58 | CP3/ HP3 |
| 41 | 16.44 | 5.16 | CP3/HP2 |


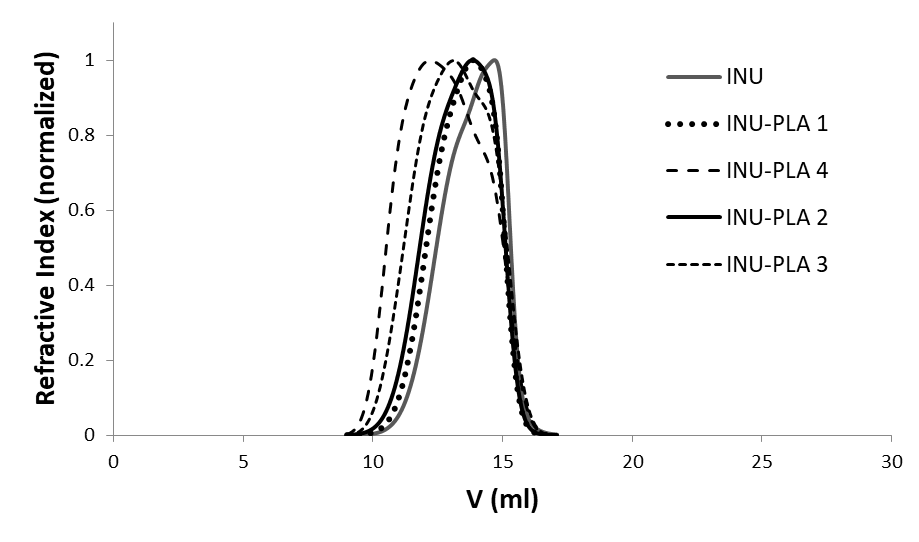


**Figure S4**. GPC traces of INU and INU-PLA_1-4_ derivatives.


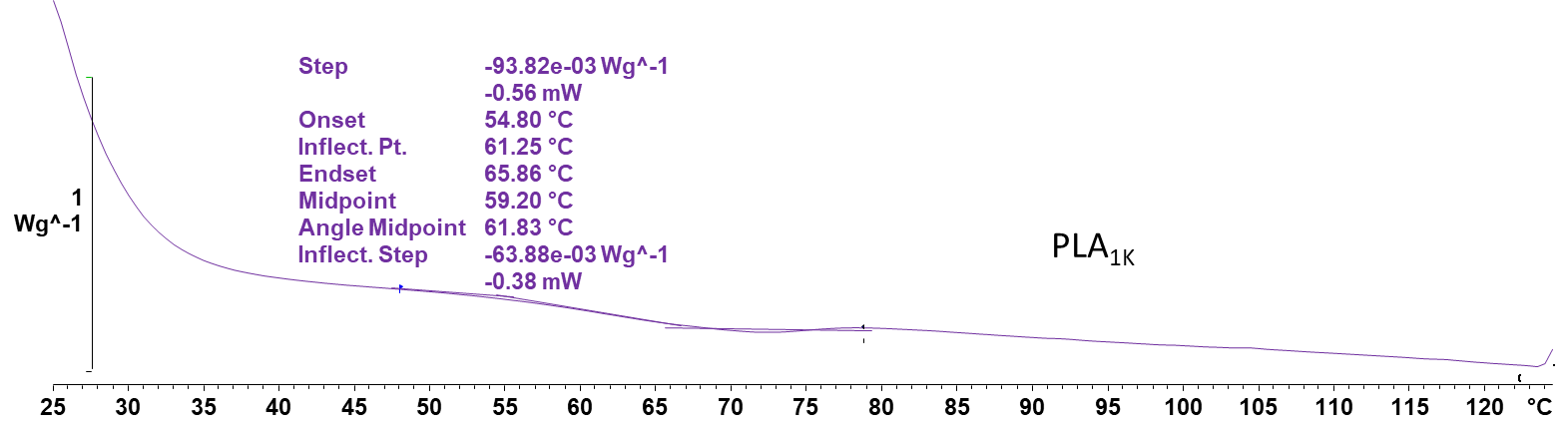


**Figure S5**. Thermogram of PLA_1K_ showing the Tg (Exo up). Recorded by DSC in non-hermetic aluminum crucible (40ul) purged with nitrogen at a flow rate between 60 and 70 ml/min, by heating from 25 to 125 °C with rate of 30 K/min.


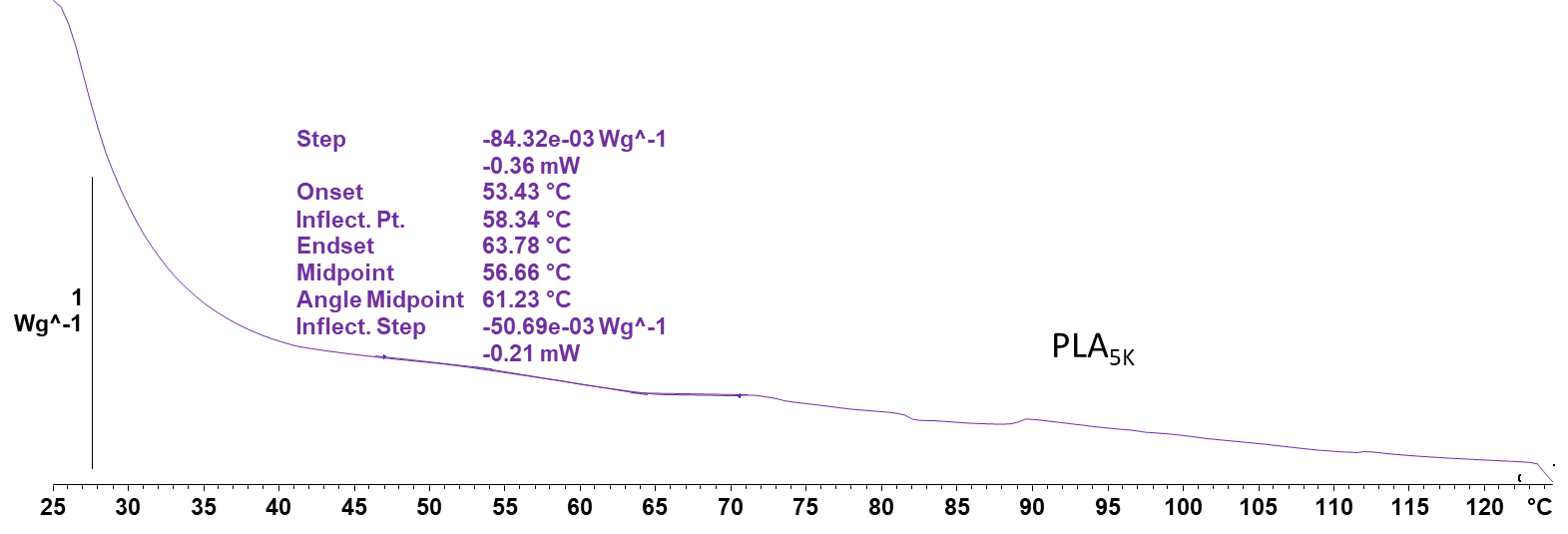


**Figure S6**. Thermogram of PLA_5K_ showing the Tg (Exo up). Recorded by DSC in non-hermetic aluminum crucible (40ul) purged with nitrogen at a flow rate between 60 and 70 ml/min, by heating from 25 to 125 °C with rate of 30 K/min.


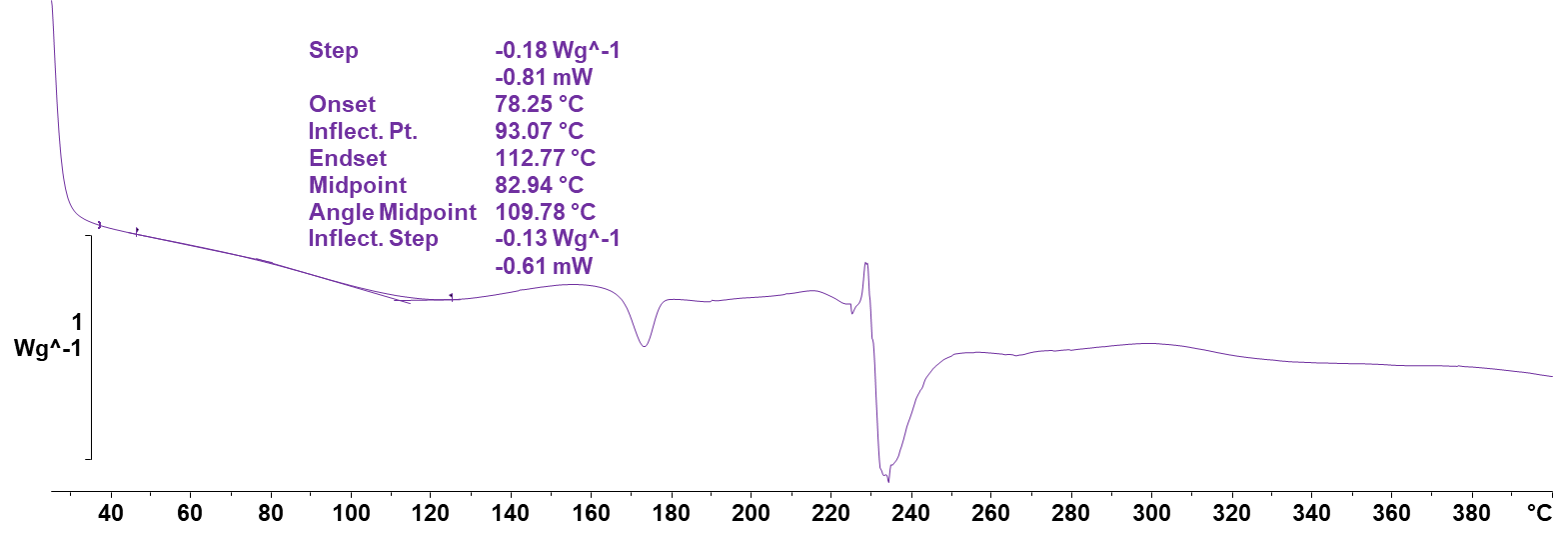


**Figure S7**. Detail of the thermogram of INU showing the Tg (Exo up). Recorded by DSC in non-hermetic aluminum crucible (40ul) purged with nitrogen at a flow rate between 60 and 70 ml/min, by second heating cycle from 25 to 400 °C with rate of 10 K/min.


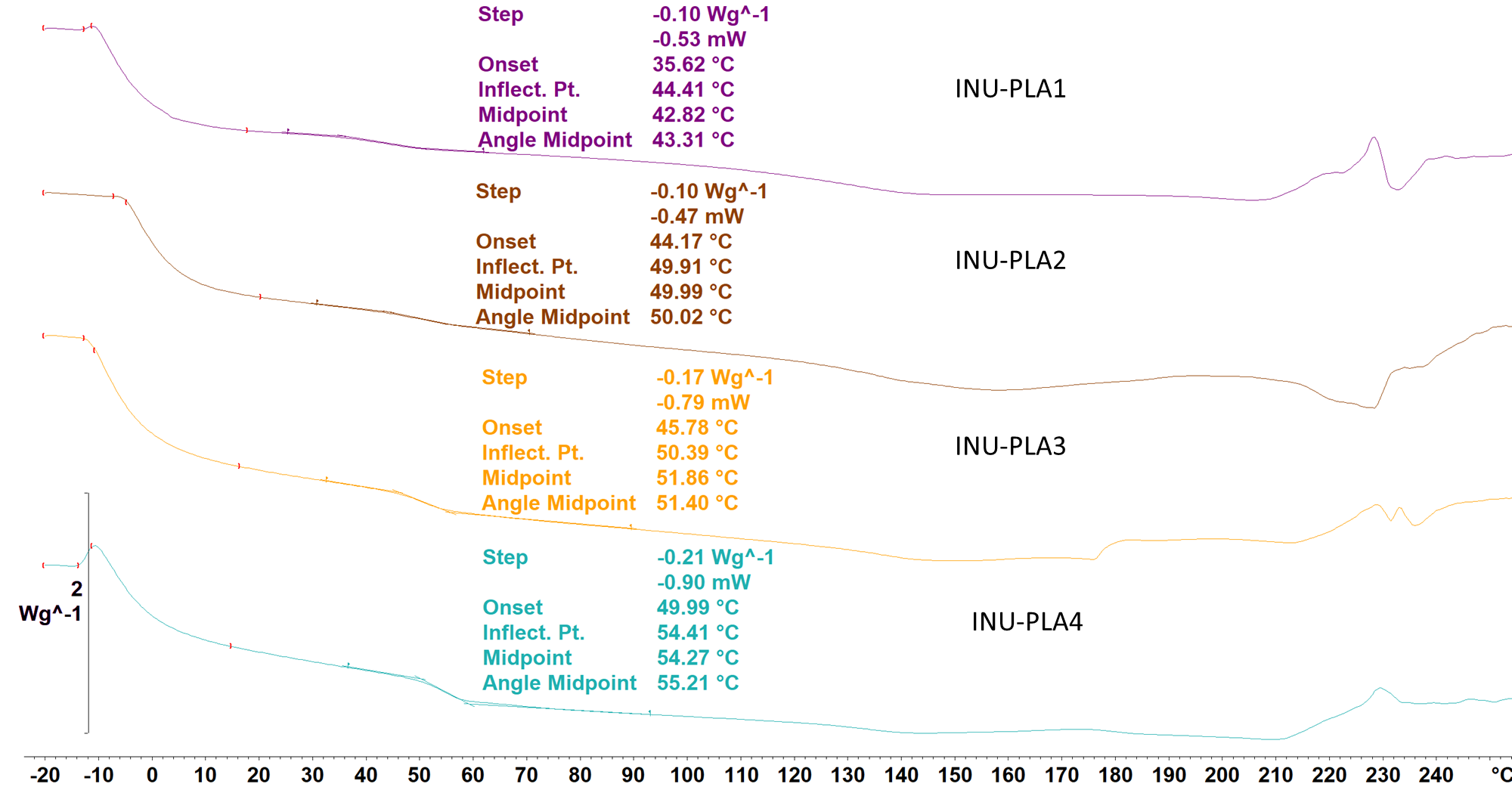


**Figure S8**. Detail of the thermograms of INU-PLA_1-4_ showing the Tg (Exo up). Recorded by DSC in non-hermetic aluminum crucible (40ul) purged with nitrogen at a flow rate between 60 and 70 ml/min. The following cycles were applied

1. Heating 25 to 120 °C, 30 K/min

2. Isotherm 120°C, 2 min

3. Cooling 120 to -20 °C, 30 K/min

4. Heating -25 to 300 °C, 30 K/min


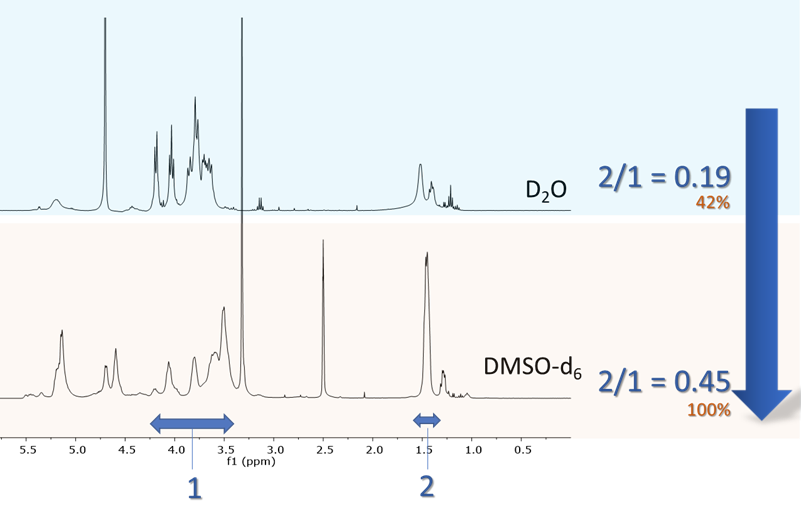


**Figure S9**. ^1^H NMR spectra in D_2_O (upper panel) and DMSO-d_6_ (lower panel) of INU-PLA_2_.


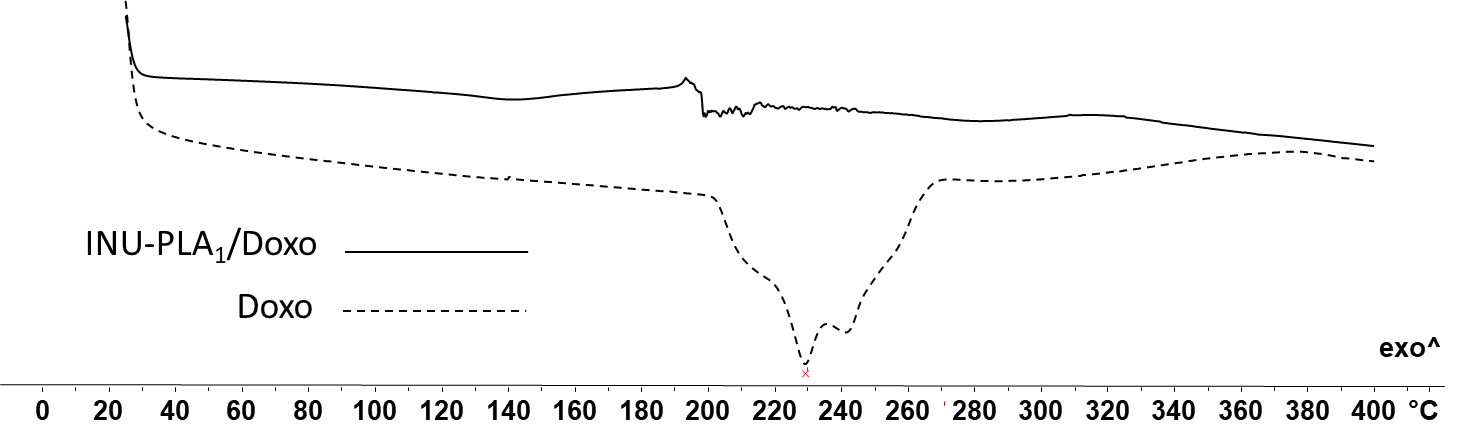


**Figure S10**. Thermograms obtained by DSC of Doxo loaded INU-PLA_1_ nanoparticles and free Doxo. Subsequent peaks in the thermogram of the drug are due to melting and successive degradation


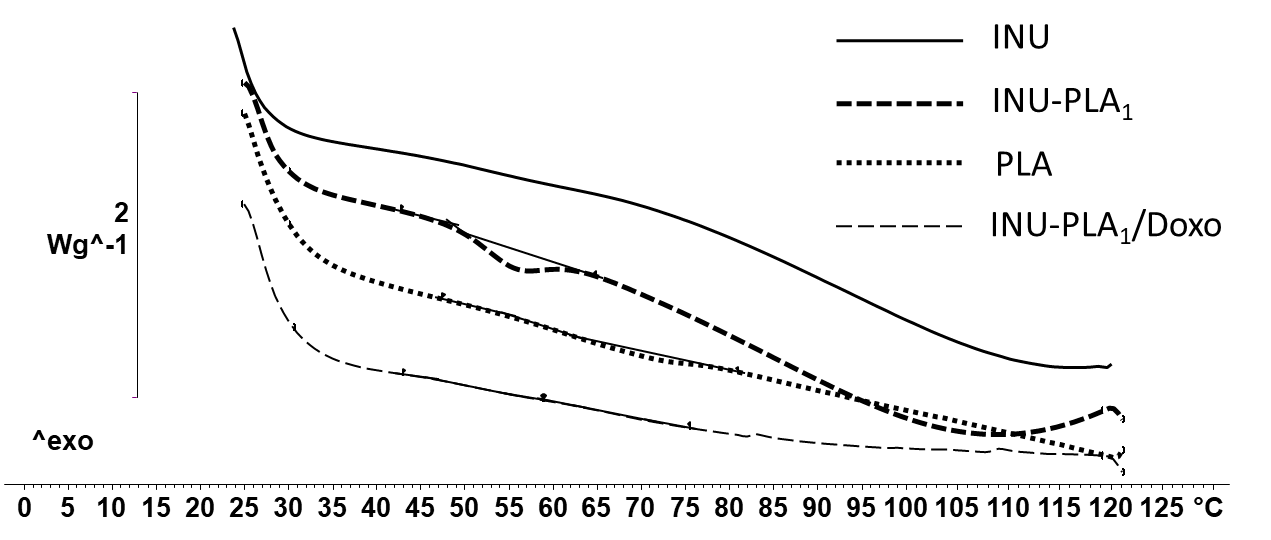


**Figure S11**. Thermograms, obtained by DSC, of INU, INU-PLA_1_, PLA_1K_ and Doxo loaded INU-PLA_1_ nanoparticles (INU-PLA_1_/Doxo).

**Figure S12.** Doxo diffusion through a dialysis 3.5KDa RC evaluated in TRIS buffer 0.5·10^-2^ M (pH = 7.4) at 37 °C.


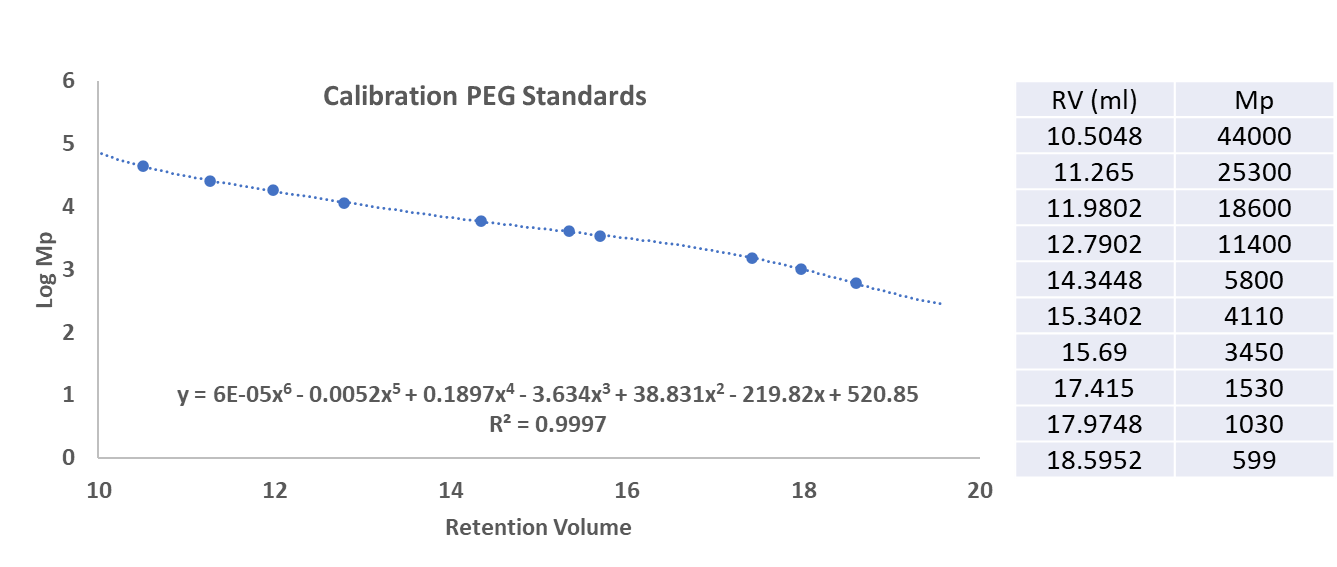


**Figure S13**. Gel Permeation Chromatography (GPC). Processing was carried out according with Gavrilov and Monteiro [1]. Calibration curve with various PEG standards (in the range 600 - 44000 Da) was obtained by polynomial regression, with satisfying correlation coefficients (y = 6·10^-5^ x^6^ - 0.0052 *x*^5^ + 0.1897 x^4^ - 3.634 x^3^ + 38.831 x^2^ - 219.82 x + 520.85; R² = 0.9997).


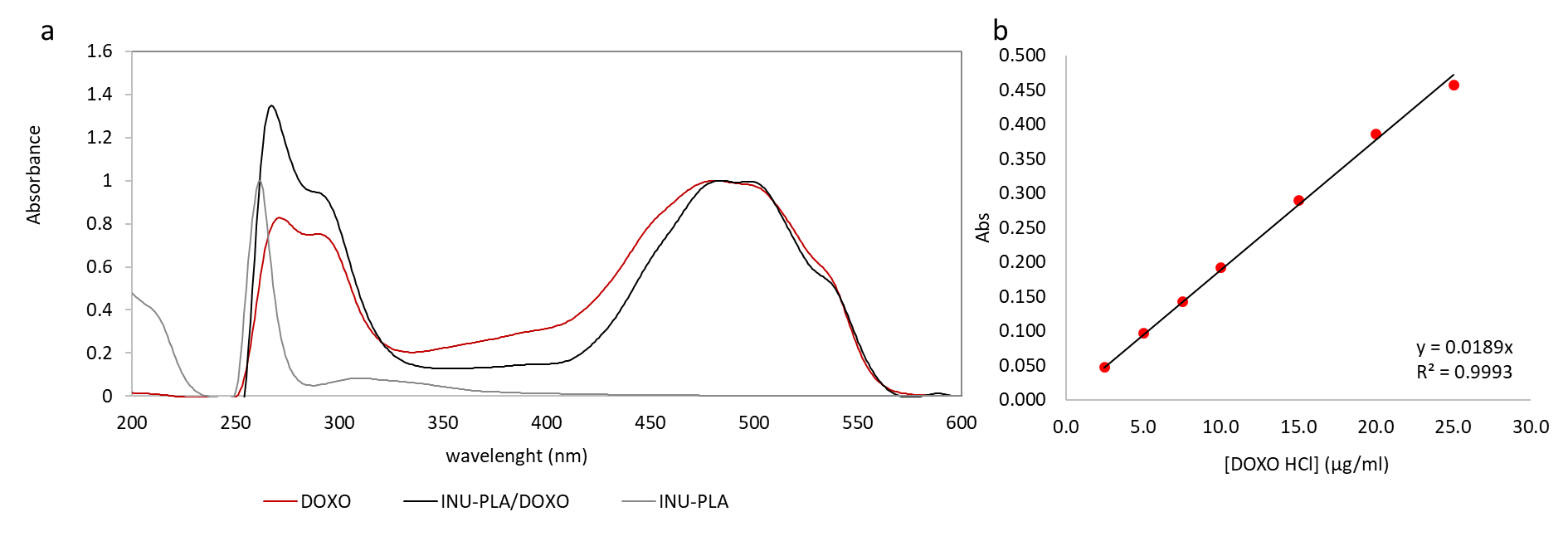


**Figure S14**. Doxo, INU-PLA/Doxo and INU-PLA representative UV-Vis spectra recorded between 200 and 600 nm (a). Calibration curve for serially diluted solutions of DOXO-HCl in DMSO/PBS 1:1 (b).

| **Table S3.** Average diameter of systems prepared by both the thin-film hydration and nanoprecipitation methods at different stages of particle preparation | | | | | | |
| --- | --- | --- | --- | --- | --- | --- |
| **Method** | **Film rehydration** | | | | **Nanoprecipation** | |
|  | Pre-liofilization | | Post-liofilization | | Post-liofilization | |
|  | **NF | *F | *NF | *F | *NF | *F |
| INU-PLA_1_ | 1115 ± 560 | 182 ± 5 | NA1 | 215 ± 7 | 156 ± 16 | 139 ± 35 |
| INU-PLA_2_ | 1302 ± 555 | 62 ± 10 | NA1 | 220 ± 7 | 147 ± 10 | 158 ± 28 |
| INU-PLA_3_ | 5636 ± 467 | NA2 | NA1 | NA2 | 170 ± 12 | 273 ± 60 |
| INU-PLA_4_ | 4247 ± 275 | NA2 | NA1 | 166 ± 8 | 230 ± 10 | 277 ± 41 |
| PEG-PLA | 840 ± 84 | NA2 | NA1 | NA2 | 996 ± 181 | 67 ± 8 |
| **Data obtained by Coulter LS 13320 (Beckman Coulter, Inc., Brea, CA, 173 USA), in bi-distilled water  *Data obtained by dynamic light scattering  NA1: samples too polydisperse for cumulant analysis  NA2: data quality too poor for cumulant analysis (too low recovery after filtration) | | | | | | |


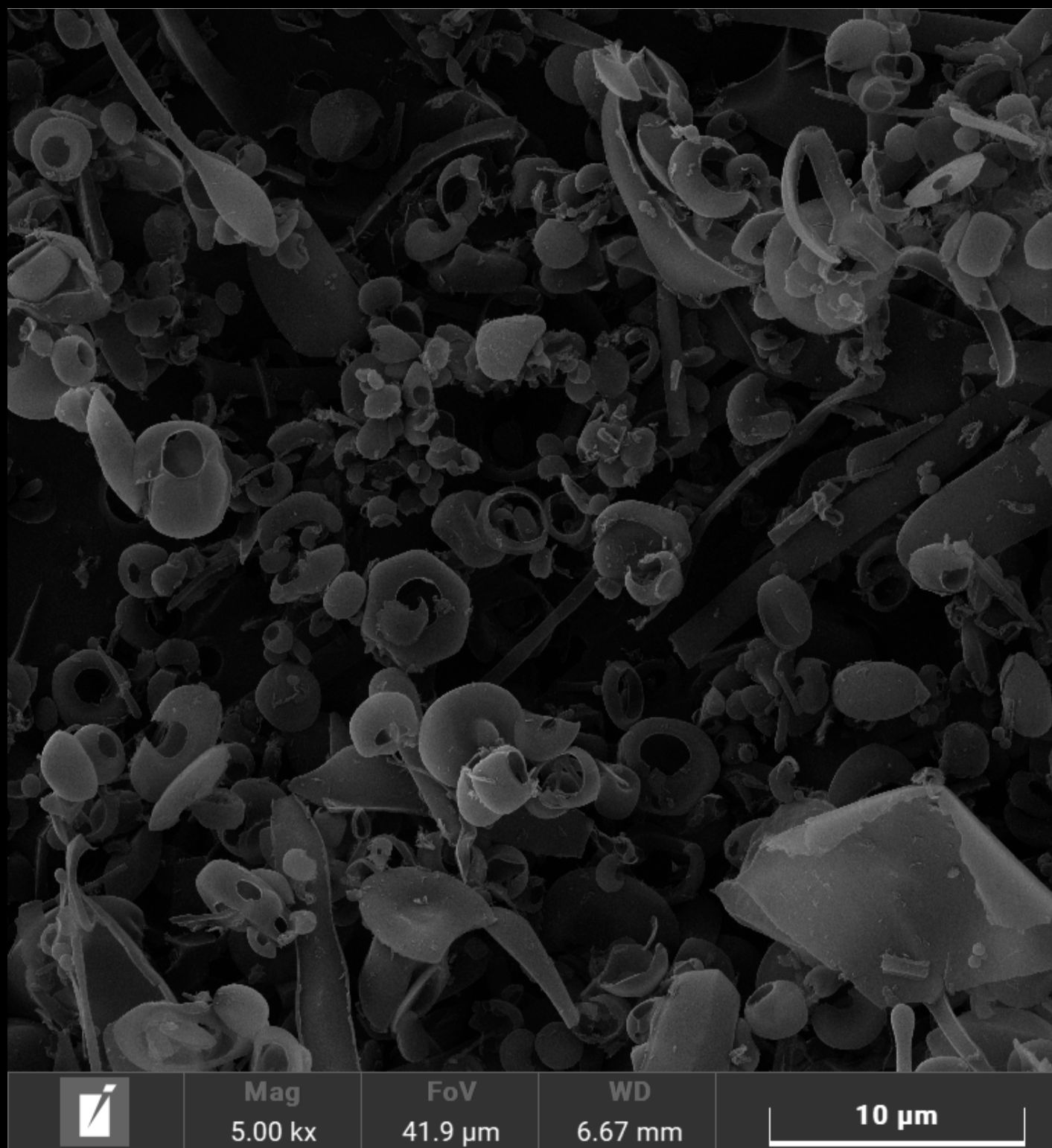


**Figure S18.** Additional SEM micrograph of INU-PLA4 after film rehydration.

References

[1] M. Gavrilov, M.J. Monteiro, Derivation of the molecular weight distributions from size exclusion chromatography, Eur. Polym. J. 65 (2015) 191–196. doi:10.1016/j.eurpolymj.2014.11.018.
